# Supplementary material for: In silico functional annotation of hypothetical proteins from the Bacillus paralicheniformis strain Bac84 reveals proteins with biotechnological potentials and adaptational functions to extreme environments
Source: PLoS One. 2022 Oct 13;17(10):e0276085. doi: 10.1371/journal.pone.0276085 (PMC9560612; doi:10.1371/journal.pone.0276085)
Supplement: S1 Fig — Networks are visualized using Cytoscape. (PDF) [file pone.0276085.s001.pdf]

Supplementary Figure S1: Protein-protein interaction networks obtained from STRING analysis. Networks are visualized using Cytoscape (v 3.9.1).

* Networks are presented in circular layout with protein of study in centre.
* Edge connecting the protein of study and its important functional partners (with highst score) is highlighted in red.
* Confidence score of the interaction is given in brackets.

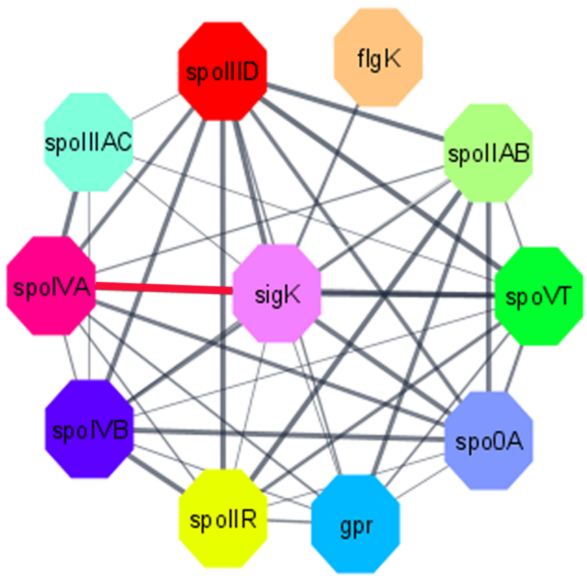

Study protein: WP_095290960.1
String protein: sigK
interacted protein: spoIVA
Has a role at an early stage in the morphogenesis of the spore coat (0.930)

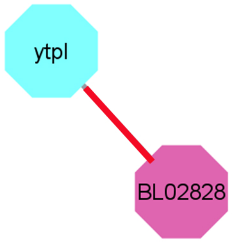

Study protein: WP_026579962.1
String protein: BL02828
Interacted protein: ytpl
Uncharacterized protein (0.930)

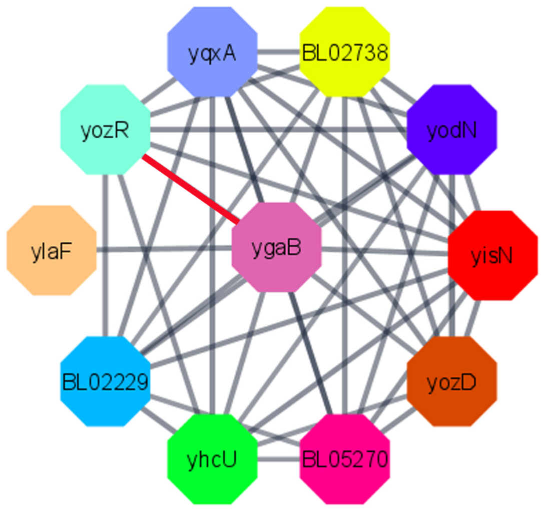

Study protein: WP_003179940.1
String protein: ygaB
Interacted protein: yozR
Involved in spore germination (0.777)

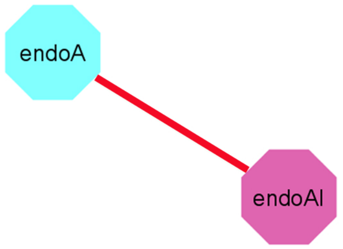

Study protein: WP_006638778.1
String protein: endoAI
interacted protein: endoA
Putative rnase (0.988)

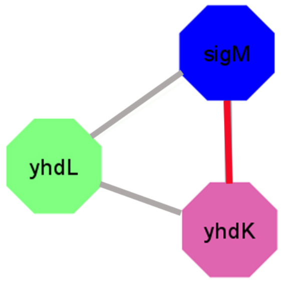

Study protein: WP_003180123.1
String protein: yhdK
interacted protein: sigM
RNA polymerase sigma factor (0.921)

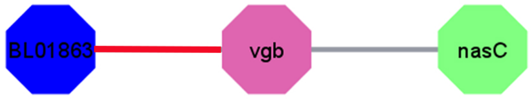

Study protein: WP_025810847.1
String protein: vgb
Interacted protein: BL01863
Putative formate C-acetyltransferase (0.722)

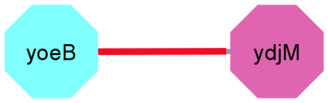

Study protein: WP_020450411.1
String protein: ydjM
Interacted protein: yoeB
endopeptidase inhibitor (0.713)

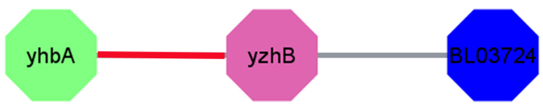

Study protein: WP_105980832.1
String protein: yzhB
Interacted protein: yhbA
4Fe-4S cluster binding putative ferredoxin (0.747)

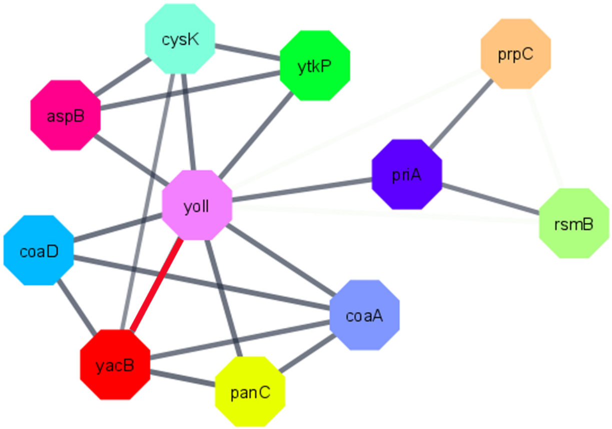

Study protein: WP_009328837.1
String protein: yolI
interacted protein: yacB
Catalyzes the phosphorylation of pantothenate (0.987)

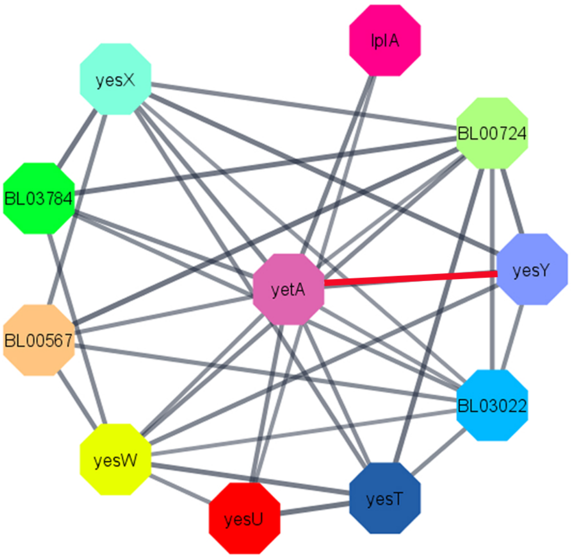

Study protein: WP_199792123.1
String protein: yetA
Interacted protein: YesY
Carbohydrate esterase (0.883)

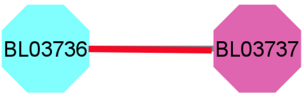

Study protein: WP_020451108.1
String protein: BL03737
Interacted protein: BL03736
Uncharacterized protein (0.845)

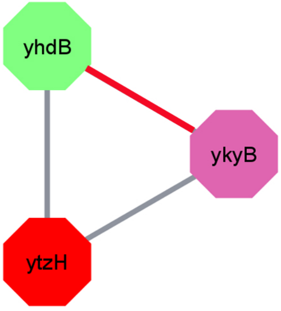

Study protein: WP_020451191.1
String protein: ykyB
Interacted protein: yhdB
Uncharacterized protein (0.773)

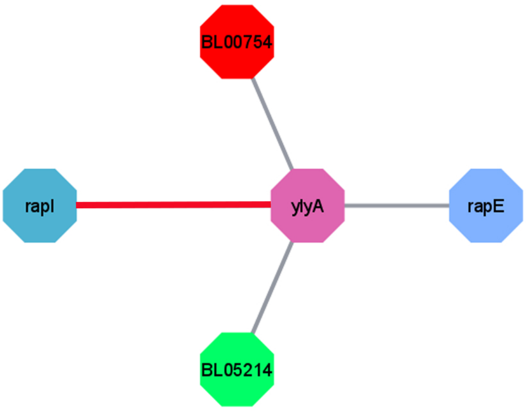

Study protein: WP_026579751.1
String protein: ylyA
interacted protein: rapI
Uncharacterized protein (0.716)

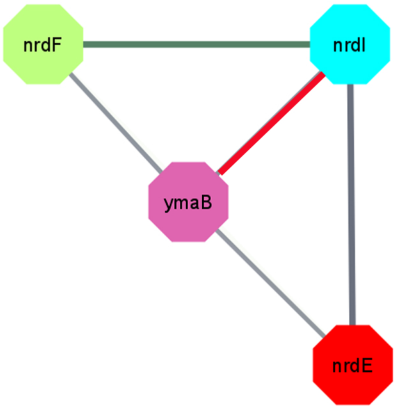

Study protein: WP_105980957.1
String protein: ymaB
Interacted protein: Nrdi
Probably involved in ribonucleotide reductase function (0.771)

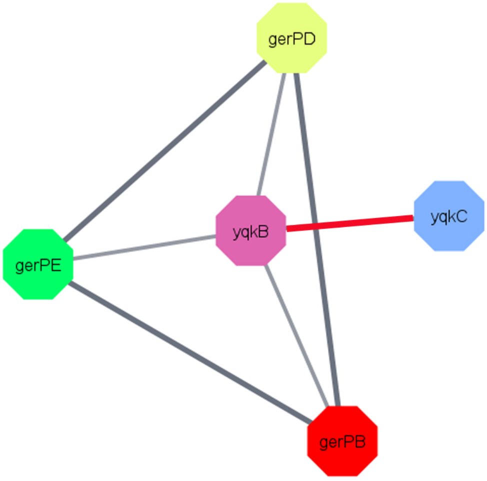

Study protein: WP_020452052.1
String protein: yqkB
Interacted protein: yqkC
Uncharacterized protein (0.866)

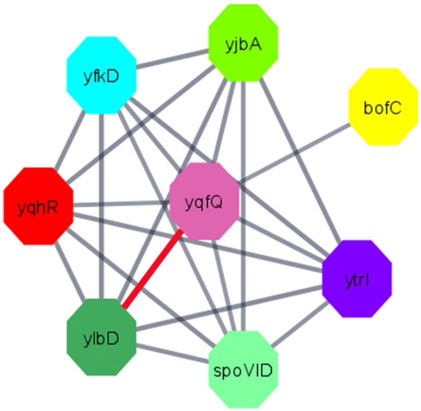

Study protein: WP_026579290.1
String protein: yqfQ
Interacted protein: ylbD
Uncharacterized protein (0.755)

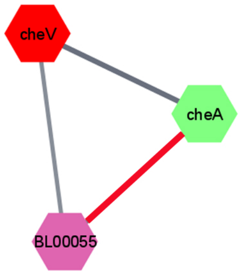

Study protein: WP_234026546.1
String protein: BL00055
interacted protein: CheA
Two-component sensor histidine kinase (0.876)

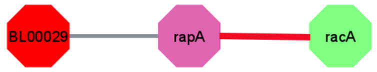

Study protein: WP_023855527.1
String protein: rapA
Interacted protein: Raca
Required for the formation of axial filaments (0.836)

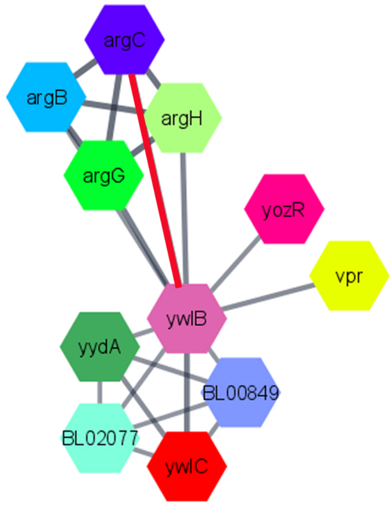

Study protein: WP_023857076.1
String protein: ywlB
Interacted protein: argC
N-acetylglutamate gamma-semialdehyde dehydrogenase (0.902)

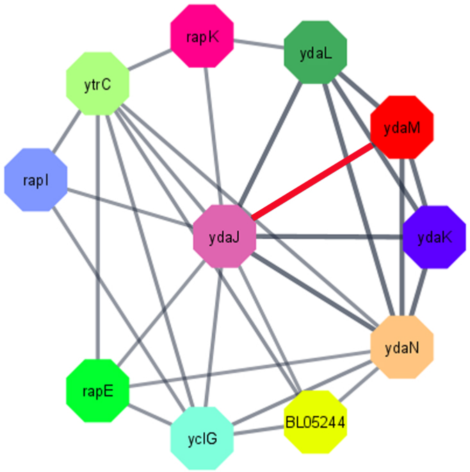

Study protein: WP_023856884.1
String protein: ydaJ
Interacted protein: ydaM
Uncharacterized protein (0.992)

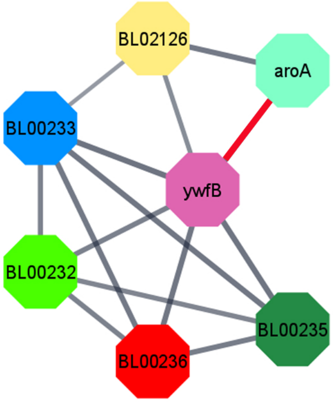

Study protein: WP_020453535.1
String protein: ywfB
Interacted protein: aroA
3-deoxy-D-arabino-heptulosonate 7-phosphate synthase and chorismate mutase-isozyme 3 (0.981)

The type of interactions are indicated with the colored edges (STRING analysis)

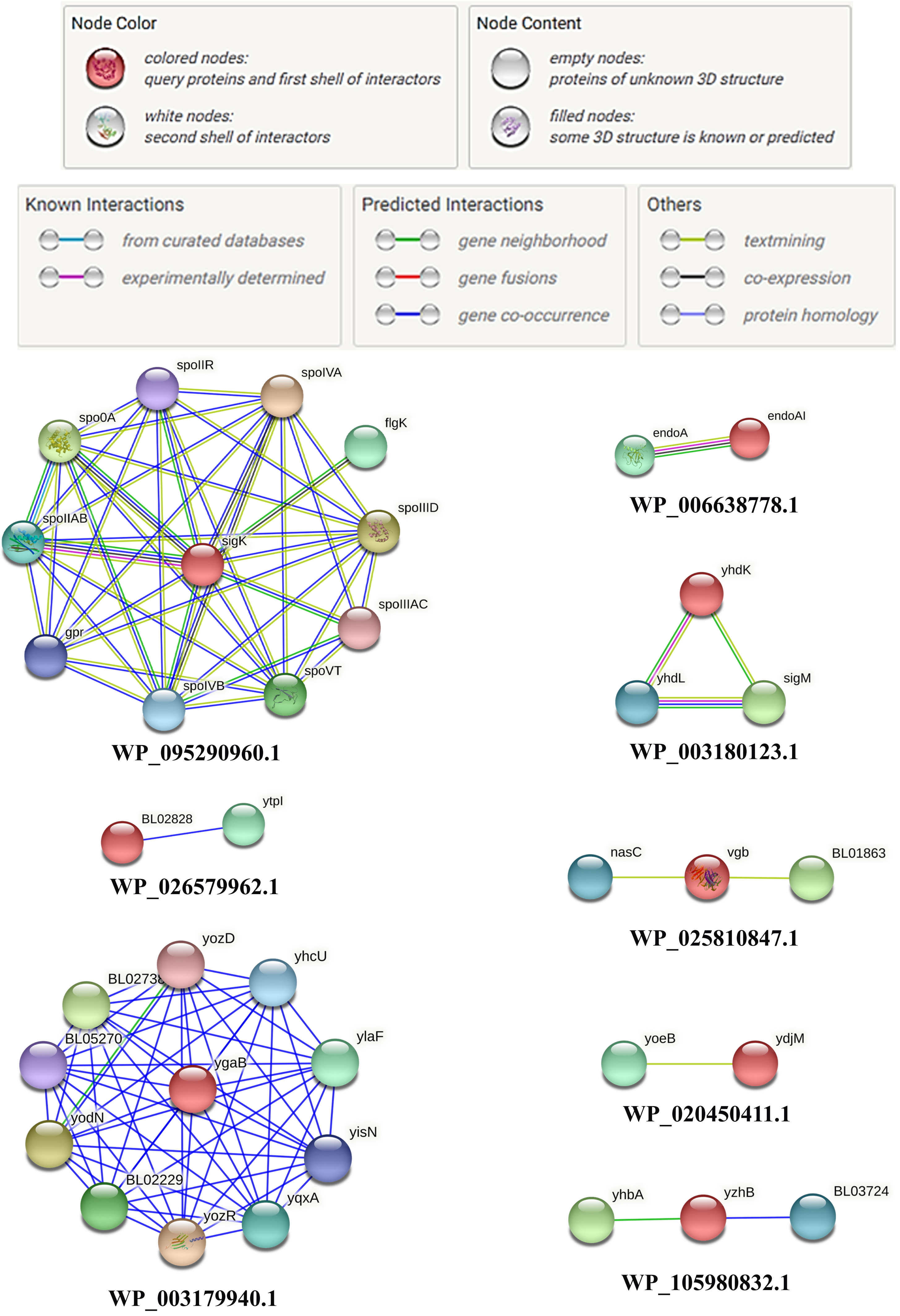

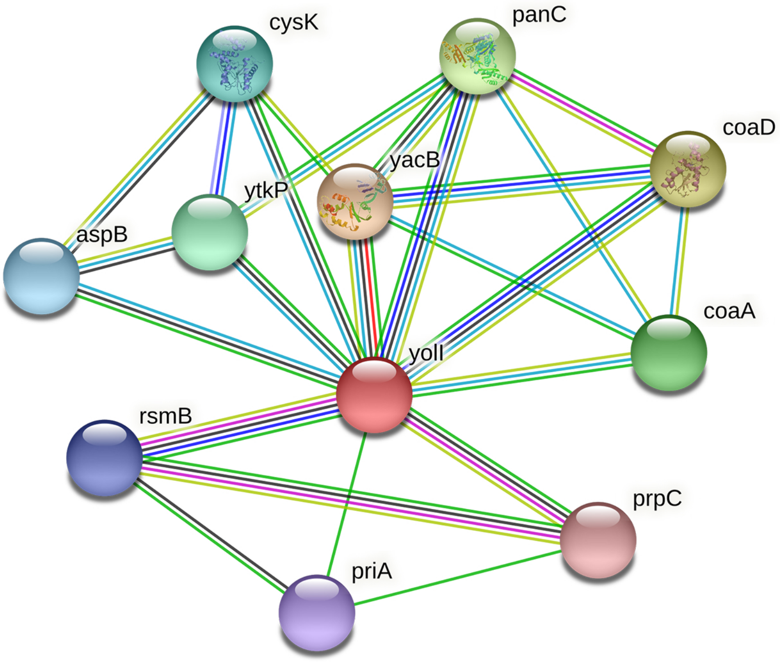

WP_009328837.1

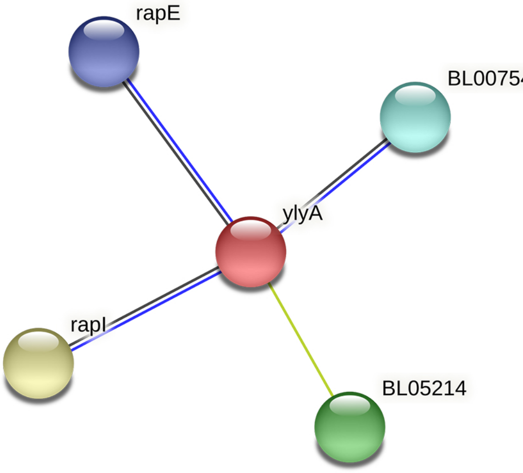

WP_026579751.1

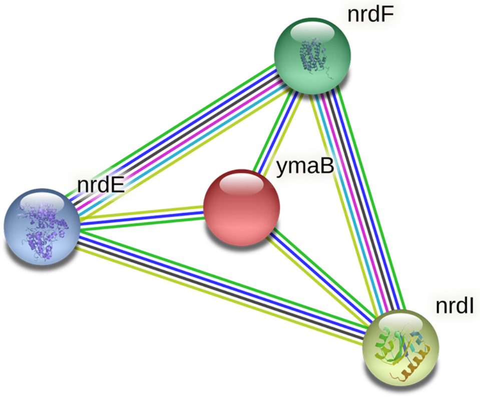

WP_105980957.1

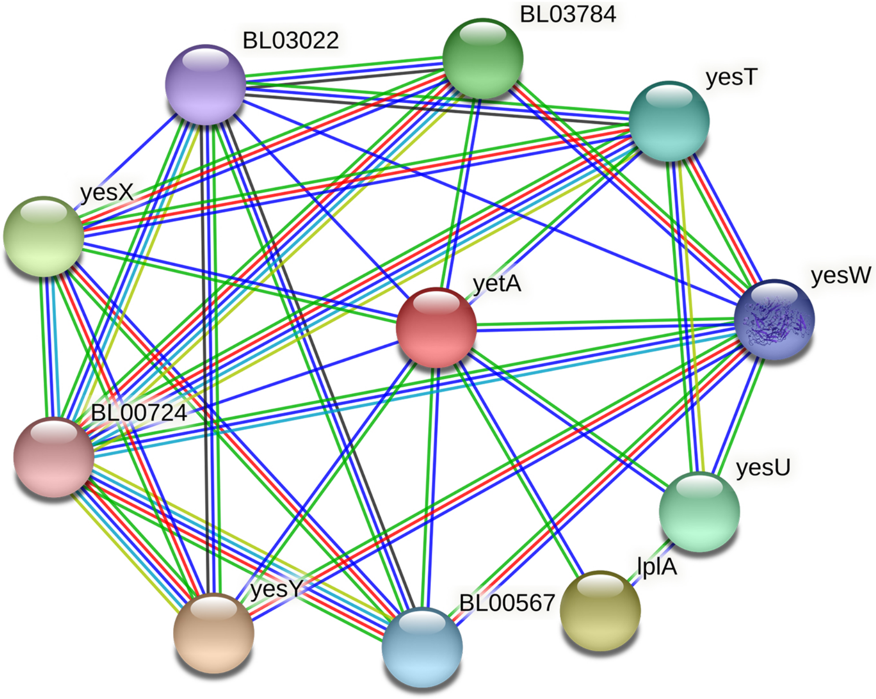

WP_199792123.1

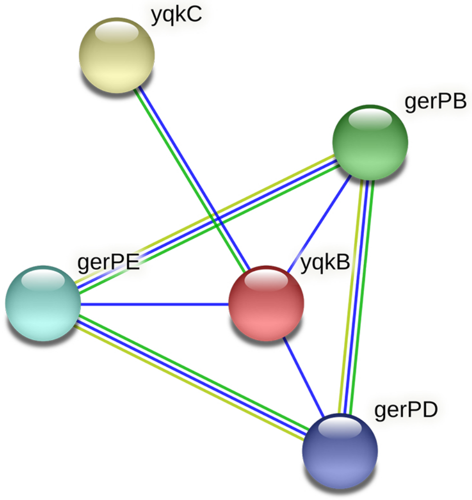

WP_020452052.1

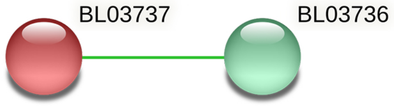

WP_020451108.1

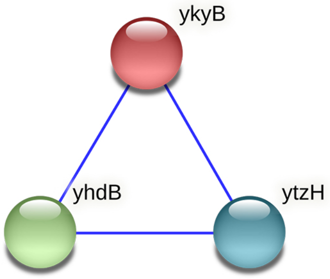

WP_020451191.1

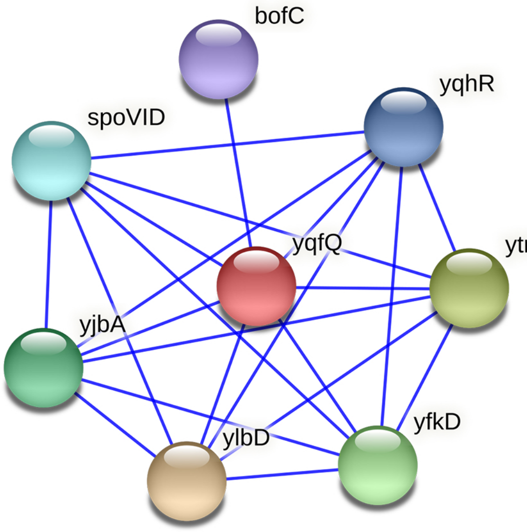

WP_026579290.1

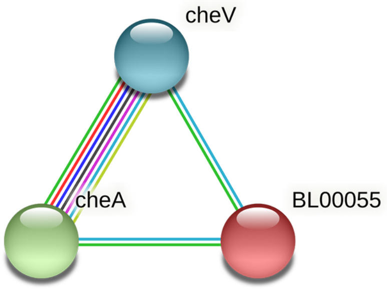

WP_234026546.1

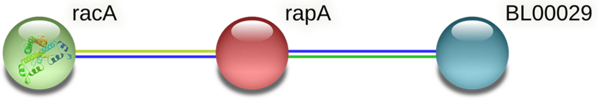

WP_023855527.1

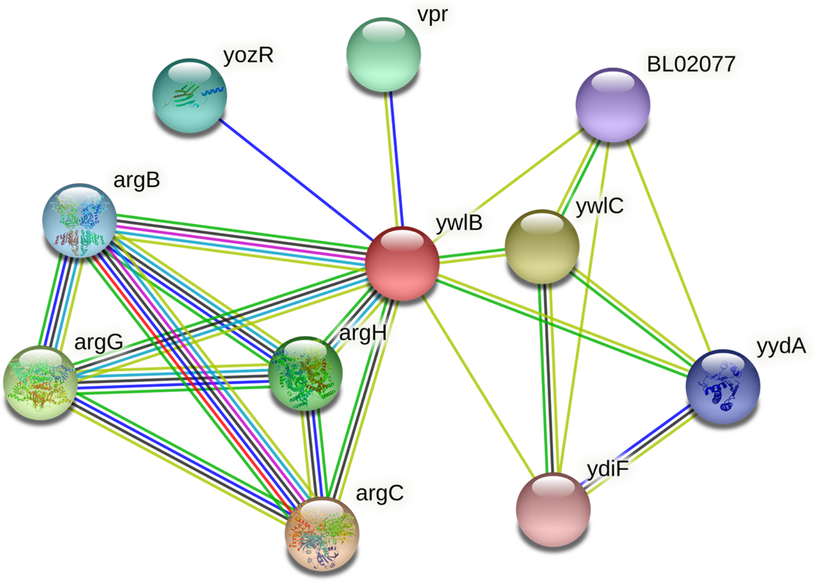

WP_023857076.1

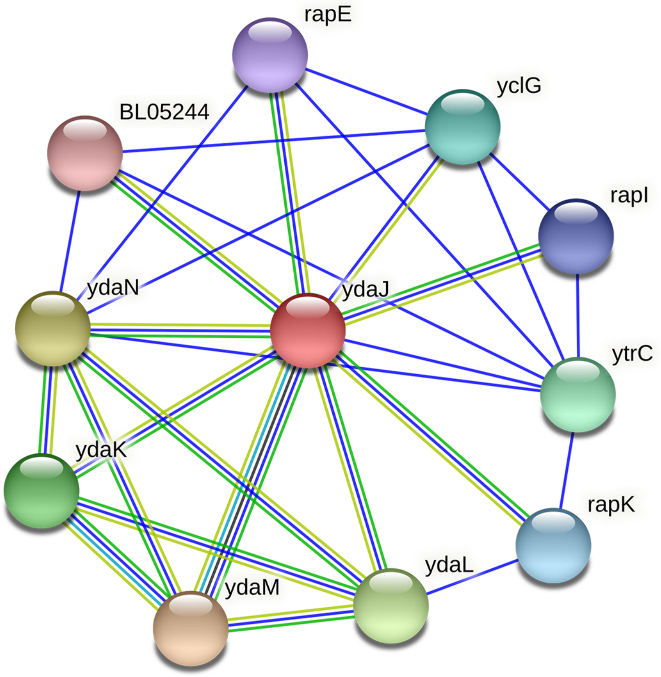

WP_023856884.1

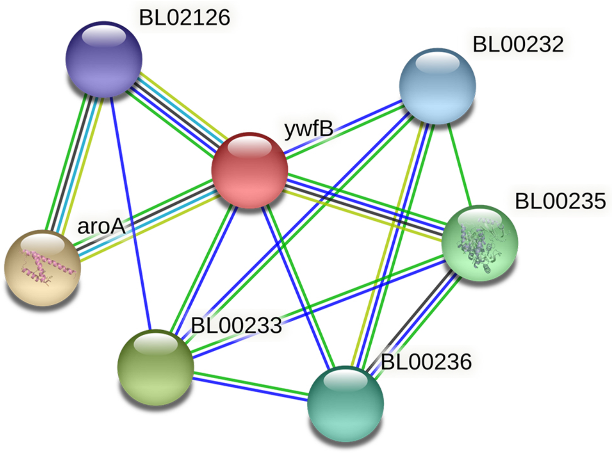

WP_020453535.1
