# Supplementary material for: In silico functional annotation of hypothetical proteins from the Bacillus paralicheniformis strain Bac84 reveals proteins with biotechnological potentials and adaptational functions to extreme environments
Source: PLoS One. 2022 Oct 13;17(10):e0276085. doi: 10.1371/journal.pone.0276085 (PMC9560612; doi:10.1371/journal.pone.0276085)
Supplement: S2 Fig — (PDF) [file pone.0276085.s002.pdf]

**Supplementary Figure S2:** Promoter analysis of the 37 proteins using BPROM

**BPROM** web tool (in default settings) was utilized to perform the promoter analysis. All the DNA sequences were downloaded from the NCBI database. The Shine Dalgarno (SD) sequence was manually assigned in this case. No experimental transcriptional start sites were found for these proteins.

**Text colors:**
**Green (Bold) – START Codon; Red (Bold) – STOP Codon**

**Highlighted colors:**
**Green = -35 box; Yellow = -10 box; Blue= - Shine–Dalgarno (SD) sequence**

**Protein-1** (WP_158700706.1)

GCGTCGAAATCGCCAGCTCTTTCGGACCGCTGGCCGGAAAGATATGGAGAATCGGGACAAT
GGGGTACAGCTGCCGAAAAGAAAATGTTCTTTTTACATTGGCTGCTCTCGAAGCGGTTCTCA
TTCGCACAGGTGCTTCAGTATCGCCGGGAAATGCGCTTCAGGCGGCTCTGGATTTCTATCAT
GAGTAGGCTAATCTC**ATG**AAAACGTTTGACTTGCTTTTGAAAGGCGCTTCCGTCGTGTGCCC
GGAAGGCGTAAGGAAAACCGATATCGGCGTCAAAGACGGGATGATTGCTGTGCTGGGCGTG
CTTGACGGCAGTGCGTCAATACATTTT**TAA**

**Protein-2** (WP_230368348.1)

TGGACCGCACCCGTAGCCGCCCATCAAATAACTACCCAGTCAAGACCCCGGATCTTCTTTTCAACGGTT
TTCTCGCCCTTAGCTCTAATCAAAATATAAAGTCATCAGCAATACAAATGTAAATAAACTACTCAAAA
AAGTTGTAACCGACAGTTCATGGATTAAGGCACACTCATGCAAGCGTGCTGCTAGATCGAAAG**GTG**TC
AATTTATTATGTAAGTGCAAGGTTAGGACACGCTGATATAGAAACCACCTCTAGCACATATGCTCACT
TAATTAAAGAGCTACGAGAAAGAGATGAAAATGAGACTGTAAACATTTTTGAGGAAATGGCTATCGA
GGAAGCTGAAATTGCA**TAG**

**Protein-3** (WP_095290960.1)

CTTGACTTAATTATATAATTTTTTATACAAAAATGAAACAAAAATATATTTTTTTATACACAG
GATTGATCATTCATTACCTTCAGTTTGCATCTTTTTATTTTGTCATCAAACAGGAACTATTTAG
ATATAGTTCGGTATCTTTTTAGTTCAGTCCCTCAACCTGGTAAAAGTAATTGCCCAAAACGG
ATAACCTGTGA**GTG**GCATTTAAATCAGCCTTATCGAGAAAAAAAGTCATCGCCGGCCGCTTC
AGCCTTGATTTGAAGAAAGAGAAGATGCAGCGGGAAATTGCGAAGGAGCTCGGGATTTCGC
GGATTGAAAAGCGCACGCTGATGAAGATGTTTCATGAGTTTTACCGGGCGGAGAAGGAGAA
GCGGAAG**TGA**

**Protein-4** (WP_026579962.1)

TGCTTGTCTGGAATCTCCAGCGTCAGATCGTTTACAGCTGTAAATGAACCGAACTTTTTCGTG
ACGTGATCGATTTTGAGCATAATAACTCCTCCTTCCAATTTACAATCATACGTTAGTCATACC
ATTTTAAAAAGGATTTTTTTGATTTTTATATAATTTTAAACAAATAGATCATATTTTGAGAAA

GGGGAAGAGAA**ATG**GAAAATTATTATGTAACCGTTTTCGATACTGGTGGAGAAACGCTTTT
GAATGAGCGATTTGAAGCGGCCGATGATGAAGAAGCGAAAGAGAAAGGAAGAGGAATGCT
GAAGGAAAAGCAATTTTTGGAGCATACACACCGGGTAGTACATTCGTCCGGTAAAATCATTG
TTTTCCGGAGC**TGA**

**Protein-5** (WP_224146215.1)

AGGTCTTATACCCTGACACGTTCGGCATAACCTTTTTCAGATTCCAAAAAAGGAAAATCATT
GTAAAAAAATGATCCTTTATGTAAAATGAATCATATGCAGAAAAAGACAAGGTTTAATTTTT
CGAATATTTAAAATCAACTATATGAAAGGATGCTGGGGGCATTGGAAGCTAAGTTGCGTTTG
AGAAAGTCGGTAAA**ATG**CTTAATGATTGGTATTGCTTTATTAGACAAAACAATATTCAAAAT
GCTACTAAACTGCGGGAAGAAATTAAAAATATACTGCCTAATATGAAAGAAAATCAAAATG
TGCTGTTATATTTTAACCTTATAGATTCACGTTTTAAACTGATGACCGAGAATTACAAAGAAT
CTGGAGATCTGTTAAACGATATAAAATCAATAGCT**TAG**

**Protein-6** (WP_095291534.1)

CCAGGCGTAATTTTATCCAAAGAAAATCCTGGCTGAAAGCAACAAAAAACACCTTCTTTGGA
AAGAAGGTGACATGGCGGATCATTCCAGTTCGGAATGCTGCCGCATCCGGTTTAATGACTCA
AATTGAAAGCTCCCTTGAGAACCTCGTTTTTTCCTAGTAAGATAAAAGAAAAAGCGCTCCTA
AAGGAGAAAGACAA**ATG**GATGAGAAACTGATTGAAAAAATGCTCGGACAGGCGCTGAAGC
AGTACGGACGCAATGTTGCAACCGATCCGCTCAGCCCCGGCGAAAAAGAAATCCTGAAGAT
GGCTTTGCAGGAAAGGCGGATCGAGGAACCGAATGAGGGGCTGCATGCCCATATCGAAGAT
GTCATCTATGATTATGTCACAAATCAAGGCTTGTTCTCCTTT**TGA**

**Protein-7** (WP_003179940.1)

AGCAAAAAAAACAGCAAAACAGCTAATCACTGAAACAGGAAAAAGCACTTCACCATCGGAT
GAGGTGCTTTTTTCTTTTCGTTTTTTGATCCGACAAAACATCCGAATCTTAGCCACAATCAGT
CAAAGGACATCCGGCATGTATAGAGAAATGTTAGTTAGGCAATAAATGGAGTAGAGCATAG
AATGGAGGATCCCGA**ATG**CCGGAATTTGAAAACTTGGTTCAGGGGCAGATGGAGATTATGG
ATAAGCTGCTGTATCTGCAAGCGGAGATTGAAAGATGCCAGAAAATTGAAAAAGAATTGGC
TGCCCTTGAAAAGAAAGCCCAGCTTTTATCGATCCGGGAAGATATATCCCAGAAGCGGAAA
GATCTTGCTCAAATTCAGGATTTATTCCAAAAGCAGACGGAGCAGGTGATTCAGGCTTATCA
AAGAATCGAAGCA**TGA**

**Protein-8** (WP_020449960.1)

GACCATGCCGTCAATATTGCAGAAGCTGTTTTAGGCGAAGAAAAGCATTAAAAACCGGGAG
TCATTCCCGGTTTTTTATTTTGTAGGCCTTTTTTATTACCATGTGACATATTTACTAAATTATG
TGTGGGAAGGAAGAACATTCTTCTTCTGTTGACCTAAATAAAGTATAATAGTTTTATTCTTTA
GGAGGTATTAAG**ATG**CAGAAACACAGAAGAAGAAACACAACGGCCTTTACGGTTATGTCAT
ATTTCACATTTTTTGCGGGGGTCTTTTTGTTCGGTATCGGCCTTTACAATGCGGACAACCTGC
AGCTCAATGAAAAAGGCTATTACATAGCTGTGATGATTTTAGTGGCGGTCGGCGCGATTTTG
ACGCAGAAAGTGACGAGAGACAACGCTGAGGATAATGAAATCATCGCGGAGCAGGAGAGA
AGACAGAATTTGTCGAGTGGTGAG**TAA**

**Protein-9** (WP_105981192.1)

GAACCAAGTGGAGGTTTCCTGCAGCTGAAATGAAAGAATTTCTACTGACATGGTTAGCCGAA
CAGCCAAGAAATTAAGCCCTACTAAATGAGGGGAGGTGAGGAATTGAAGATCAAAACCAAA

GAATGGCTTGCTTTAAGCGAGGCGGAACGTTTTATGAAGATTTATCAAGCATTTGCAAAATC
GAAAGGAGCGCAAGT**ATG**AACCTTAACAATTTTCTAAAAGCTGACCGCGAAAAAGCGGAAA
GACTATTTATTTCAACGCGAGACCTTATTTCCGAATTGCCCGCCGCTATCGAAGACCATGATT
TTGAAGGATGTGTAGAAATTGCGGCGACAATCATTTCACATTGCAAAGACCTTAAACGAATG
GAGCACCCGGAACAGGTTGTTCGGCTTCGCGAAATCGCGTCCAAATTCGCGGGTAGGGGGTT
AAATGTTTCAGCCGTAGGGAGACCGTTTCAA**TGA**

**Protein-10** (WP_020453622.1)

CATCCCGCAAAGTCAATCGGAAATCCGAACATTAATTTTAACACCAAACACATTTGTTCGTT
ATTATAAAGCAAATTTCATATATTTTCTTTTTAATAGACAGTCTGTAAGTCAAATCTTAAAAA
TAAGCGTTTTTCTTGGACAAAGACACATAAAAAACCGGCAACACAAATATTCAATAAGTAA
GGAGGGAGATGCCG**ATG**GAGACGGTATTAATTTTTGCATCTGTTTTATCGCCGATTATTTTG
GCGCTGGTCGAATTGGTGAAGAAGACGTTCAAGGTGCCGAAAAACTTGATTCCGCTCGTTTC
ACTCTTGATAGGTTTGCTGATCGGGGCCGCAGCATATCCTTTTACAGACCTTGAACTCGTGCT
CCGTTTATGGTCCGGAGGACTTGCCGGATTAACCGCAACGGGGTTATTTGAAATCGGTAAAA
ACCGCAATGCAAGAAAAAAGAAAAATCCC**TGA**

**Protein-11** (WP_006638778.1)

GTATGATAAGTTCAAGGGTGCCCCGTATGTTTTTGGAAAATGGGAGTATAATGGAAATAAGG
AATCCGATCTTGCCTGATCAATCCTGAAAATTGATGAATTAGCGGAAAAACAACTTTGCTTG
CGAAAAGAATAATGATATCATTATGGATGGAATGGATAGAGTGTTGTATCCGTAAGTTTGGT
GGAGGTGTATGTTT**TTG**TCTGAATCCAGCGCAACAACTGAAATATTGATTCGCTTGCCAGAA
GCTTTAGTATCAGAACTGGATGGTGTCGTCATGCAAGATAACGGGAGCAGAAATGAACTGA
TTTACCAAGCCACAAAAATGTATTTGCGCGAGCGCAAAAATCGACAAATTCGGGAATCGAT
GAGACGCGGTTATATGGAGATGGCCAAGATTAATTTGAACATCTCTTCTGAGGCTCAATTTG
CAGAGTATGAGGCTGAAAACACAGTAGAGCGCTTAGTAAGCGGAGGA**TAA**

**Protein-12** (WP_003180123.1)

CTTTCCTACATGATCAAGAACAAGAAATGGGCTGAAACCATTTCAGGCAGGAAAGACCTGA
AGCTTTCCAAACGGCTTGACTATGTGGAACAGAATGGTGTGAACGTGTATGGAGCCGTCATT
ACCGGACCGTCAAAAGAGGTTGAACGGCTGATCAAAAAAGGGCCTGTCAAAGCAGCTAAAG
TAGGTGAGGTGGAATT**ATG**GAACTGGTAAGAATCTTTAAGGAGCACAACGTATTTGGATGG
GTGTCTGTCGGCACGGCCATTCTGTCCCTGTTCGTCTTAAACTTGGCCATTTTAAGCGACACG
ATCGCATATTCGTATAAGATGCTTCCTTTTGCGATGGCCGCCGTTCCGCTCGGAATTATCGAG
CTGTTCATAAAAAAAGGACGGACCGGTCTCGGATTTCTCGGCGTCGTCCTGAACATTTTCAT
TCTCGTTTGCGTATGGACCATCGTCTCCATCGATACGAATCTGCAGCTCGGCTTT**TAA**

**Protein-13** (WP_025810847.1)

TTATTGAACCATATATAGAAAAATAAGTTTATGCTTTAAAACAATCTTATGATACTAGAGCA
TGCCTGTACAGTTTATATTTAATGGAACTTTCTGTTTCACTGCATGCTCCGCCTGAGGTAACA
TGAAAATGAGAAATGCCTGGGAGCGTTCTGAAAGATCAGATGCTCAAGGAAAATGGAATAA
AGGGAGGGTCGCTC**ATG**CAGATTCGCATGACTGAATATCATGTTCCCAACCCCGAGTCGGG
GCCGTACGGGATCGCCGTATGTGCAAACGGGCGCATTTGGTTTACTGAACAAAAGGGAAAC
CGGATTGGTATGCTCACAGAGTCTGGAGATATTACCGAATATACGATTCCGACGAAGAGCGC
AGGCGCCTCGATTATTACTCCCGGGATTAGCGGTGAGCTATGGTTTACGGAGTATAAAGCGG
GGAAAATCGGAAAAATGACGCCTCAAGGGGAAATAACCGAGTATGCCCTTCCTCTAGGTAC

TGCCCCGTTTGGGATCGCCGTAGGCTGTGACGATGCCATGTGGTATACCGATATGGCCGGAC
ATCAAATCGGCAGGCTGTCATCTTCAGGCGAAATCACTGAATATAAACTGCCCAAACCGGG
AGCCTTCCCGTCATTTATTACAAGGGGAGCAGACGATGCACTCTGGTTTACACAAAATCAAA
GCGGCTCCATCGGGCGAATAACGGCAGATGGAGACATCAGCGAGTACCCGCTTCCTGGAGA
ACAATCCGGACCGGTCGGAATCACTGCAGGCCCGGACGGAGCTTTGTGGTTTACAGAAATA
AACGAAAATCAAATTGGGAGAATTGCGGTCTCAGGGAAGATCAGCGAATACCCGCTTCCGA
CAGCCAATGCCCGCCCGCACGCGATTACCGCCGGCGGCGATGGTGCCCTGTGGTTTACGGAA
TGGGGTGCGGGTCAAATCGGCCGCATTACAGTCAACGGGGATATTACGGAATATCCAATTCC
GACGGCCGAGTCTGAACCGCACGGGATAGCGGCCGGACCAGCCCAATCGATCTGGTTTGCA
GAAGAGTGCGGCCGTATTGGGAAAATCTCTATTCTTGAT**TAA**

**Protein-14** (WP_020450411.10

GTGATCATCCTATTCATTTTGGTTTCTCTTTTATATACGAACGATCCCGGCAAAGGTGTCATC
TGTTCTTGATAGTCTGGTAGAAAACTGGGCGTAATAATTGCATTTTAAACGTAAACAAACTG
TAAAATACCATTTCTATGAAAAAAGGTATAATTTGAATGTCAGAAAAAAATCCCATTCACGG
AGGATATTTTTAT**ATG**AAGAAGATACTTTTTTCTGTTTTTGTTTTGGCAGGCAGCTTGCTATT
GGCATTCAATTTTGCCGGTGAGGCGAGTGCTAAAACGAAAAAGTCAGCGGCTACATCACTT
GGTACAACGGCGTGGGCAAAGTCGGCGCTGACGGCAAAAGGCTCGGCCATTGGGACTGCGC
CACAAAGATGGGCTTCGATGTTCCGAGAAAAGGCACAAAAATAAGAGCCTATTCTAAAGCA
AAACCAAAGAAAGTCATTACAGTCTATAAATATGATGTCGGCAGAATGCCGGGCGCTGTGTT
GGATGTAAGCCCGAAAGCTTTTAGAGCTTTGGGATTTCCGACAAGCAAAGGAAAGGTAGCC
GGACATTATACATATAAAAAA**TAG**

**Protein-15** (WP_105980832.1)

GTTCTCAATCAATCGTTCTTTTAATGTTTCAAAAGTATCTGCACTGGCGAACCGGATTTTATC
GATGCCGATTTCCTGTGCGAATTCGATGACATCCTGTTTCAGTTTTTTCACATCCATCGTGCA
GAGCCTCCTTTCTTTGTTTTCGTTTTCGCTCTCTGTATAATAGAGTGTACCATGACACGGGAG
ATGAATTTAAA**TTG**GAACTTCGTTTGCAAATCGCGCCTGCTATTTTAAACCGCATTCCTTCAT
TTAAGGTTGGTGCTGTGTTATATCAAGATATTGAGATCACCGGCTCCCCGCAAATGCTCAAA
GGCCGGCTCCGCCTTTTTCAGGAATCGCTTTTTTTTGACTATGCCGATCGCAGTCCCGAAGAT
GAAATGGCCGTTAAAGAATGGCATGATGCCTTCCGGGAGCTGAACCCGTCTTATTCAGGACG
GGAAAGCGGACTCGAACGGATGCTGAAAGATATCAAAGAACAGCGCTTTGTTGAGCCGGAA
AATTCAGCGATTGATCTGAGCCGTTTTTTCTCCCTCAAATATTTGATACCGGTTCATATATAT
GATGCCCGGAAATTAAACGGTCCGATTAAAATCGAACCCGGAAGTCCCGGCGAGATCATCA
CGTTCTCCGATGATGCTGGAGCATTCGGCACGATCACCGGAAACCTCAGCCTTCATCATGCA
GGAACGGATACAAAAGATGCCTTGCAGCTCGTCTTTTTCAGGCCCTCATTATCTATAGAAGA
ATCCCGCCGCCTGCTCGAGTCTTTAACAAAAATGTTCGAACAAATTCACGGCGGCGAACATA
TCTCAACCGTCTTTCCT**TAA**

**Protein-16** (WP_009328837.1)

GGAATTATATGAAGAAAGGCTCTCACTTTGGCTTTCCGGAGGAGCTAATGAAGATCCGAATT
GCACTAAATGCTATTTTAGACCTGCCTGTCAAGGAAATGCTTGCCCTTTAGAACGGATTGAA
GCGGAAAAAACTCCATGTCCGCCAATAAAACAAAACCTTAAGAGATATCTAAACCTTGTAG
AGGAGGACTCCATTT**ATG**GGTAAAAAAAAAGATGTTGTTAGGGGTTACAGGTACAATTGGTG
CAATCAACCTTCCTCAATTTATCTATACTTTACGCGACGAATATGACATAGATGTTATTTTGA
CAGAAAATGCTCAACGATTCCTATCTCCGCGAGCTATAAGGCCTTTTGTTCAAGGAGTTTAT

GAGAATGTTTTTTCTGATTGTTCTTCTAAAATCTTACACGTAGAACTAGTAAACGAAATAGAT
CGTTTCCTTATACTTCCTACTTCTGCGGCTACGCTTTCAAAAATAGCTAACGGAGCTGCCAAT
GACCTTTTAAGTTTAAGTGTACTTAATTATTCAGAGGCTATATATATTGCTCCAAACATGAAC
AATGAAATGTGGAATAATCCTGCTGTACAAAAAAATGTAAAAATTATAAAAGAGTTCGGGC
ATATTTTTGTTAATACTACCAGCTTGGGAATAAAAGCATCAAGTGGGGAAATTGTTCAAACT
GAAGCGGGGCTTCCAGATCCTGATGAACTTTTGAAATTGCTAAGTGAAAAAGGAACCGTTTT
ATCAAAAAGC**TAA**

**Protein-17** (WP_003180732.1)

TATATGACTTCCCTCTCCGACGAGGAATTTTTGACGAAAAAAACAAAGCCGTTTTACCTTGA
TGAAGGGATGACCCAAAGCCGCTGGCTGACGGAAACGCTCACCCACGTCTTCCATCACAGG
GCCCAGCTGTTTAATTATTTAAAACAGCTCGGCTATGACGTCAACATGTTCGATCTGTATCTT
TAAAAGGTGTTCAT**ATG**AAGCTGCCTCTGACAAAAAAAGAACGGTCAAACTTAAGGACAGC
CAAGGTAAAAATAAAAAACACGGCATATATGGATCTGTCCGTCCTTGCACATACGCTCGGTT
CGTCTTTGGAGCGCGCCGCATACATCAAGGCGCTCGCCCAGTTTCAAACCGCTCCTTCCATC
GGGCCGAAAATGGCGCAGTGCGCCGTTGACCTGGGCTACTATTCCCTTGGCGACATCAAGCA
TGAATCAGGTGCGGATTTGATCATCCGCCTCGAAAAACTGAAAGGCTACTGGGAAGATCCTT
GTGCAGAAGATGCGCTGCGATGCATCGTCCATTACGCCAACGATCCCGAAAGCGCTAAAAG
CTGGTGGGATTTCACAGAGGAAAGAAAAATGTACCGCGAACGCTGCGGATACCCTCCGGAT
AGACCGTCAACGCCCTGGTACGAAAAAAAACGGACA**TAA**

**Protein-18** (WP_199792123.1)

TTTGTTTTCAGAGAATAAATGAATCGCCGCAGGCGCTTGCCCGCGGCTTTTTTGTTTCGTTTT
GCTAAAAAAGGGATGATGCCTGCAAAAAAAGGAGCAGACACCTCTCCAATATAAGCAAAAA
AGGAATATTGACGGTCAAAAGGCTAAACAGGAAAATATTCACTGACCAATCGGTTAAACAG
AAAGGAGGAACACTT**ATG**AAAGCACAGACTGAACTAAAATGGCTGAATAGGGCGCCTGCCG
CGTTCGGCGGGGTGACCTGGGGAGTTCCTTGGGCAAAAGGAGTGCTGCAAAAAGGAGAGAC
GGTCGCCATTGCTTCGAAAAACGCCCCGTGTTTGCAAACATGGCCGCTCGCATATTGGCCTG
ACGGCTCGCTTAAATGGACGGGGCATGCCACAGTCTTCCCTAAAGAAAGCGGCAGCCGATTT
ACATTGAAAAAAGGGGGGGCAGGCGTCCCTAAAACCGTCATGAATGTCAGTGAGCAGAAAG
ATGAAATCACGGTGAATACGGGCGCGATAGTCTGCAGGCTTGGAAAATCGGGGACGGAACT
CATCCGTTCGATTTCCATTTCCGGGGAAATGATGGCGAAAGAAGGGCGGCTCATCGCTTTAA
AAGAAAAAAGAAGCGGCTCAGATTCACAATGCATGTATCATATTGAACGGTTTGAGAGCAT
GATCAAACGTACGGCAATCGAACAAAACGGTCCGGTTAAGACAGTCGTCAAGATTGAAGGC
GTCCACCGCGGCGGAGGCATCGATGTTTGGCTCCCTTTTACCGTCAGGCTGTCTTTCTATGCC
GGTGATCCGTCCATCCGGATCGTGCACACGATCTTCATCGACTGTGAAGCAGAAACAGCATG
GATCAAAGGACTCGGTTTCGAGATGAGCGCGGTTTTGAGCGGAGCGCCGAGAAACAGGCAT
ATCCGCTATGCGGCCGACGGAGGGATTTACGGAGAGGCTTCTCAGCTGCTTGTGACGAGAA
GATTCAATGAAACAAACGGACTCTATGAACGACAGATTGCGGGACAGCCTGTACCGGAAAA
GTCGGCAGTCGTGCGCGATGCCGAGAGAAACGCCGTCTGGAACGATTTCAAGCTCATCCAG
GATTCCCCTGTTCACTGGCGCTTAAAAAAGCGAACCGGCAAAGGCTGCGCCTGGGTGGACG
CGATTCATGGGGAGAGAGCGAAAGGGCTGATGTATGCAGGCGGGGAAAACGGCGGCATCG
CACTCGGGCTCAGGAACTTCTTTGAAAAATATCCGTCATCGCTTGAAGCGAAAGGTCTTGCC
GGCGGGGAAACGAAATTGGCGGTATGGTTTTGGCCTCCTGAAGCTGAAGCGATGGATTTCCG
TCATTATTCAAAAGACACTCATGTTGAAAGCGCATATGAAGGGTTTGCCGAAATGAGGTCAA
CGGCCGTCGGCATTGCCAATACGAGCGAAGTCAGCCTCGCCTGTTTTGCAAATCCGCCGATC

GATGAAGAGCTGAATAGAGCGGCGGACGAATGGCAAAATCCCCCGCTGCTTATTTGTGAGC
CTGACTATTATTATCACACCCGCGCGCTGGGCGTTTGGAGCCTGAAAGATAAACGGCACCCG
CTCTCGGCTGTGCTTGAAGATCAGCTTGACGAGGCTCTCAAGTTTTACATGAACGAAGTCGA
TCAGCGGAGCTGGTACGGCTATTGGCATTACGGTGATATGATGCATACGTACGATGCCGTCC
GGCATGTATGGCGGTACGACCTTGGCGGCTATGCATGGCAAAATACGGAACTCGTTCCGAAC
CTCTGGCTTTGGCAGTCTTTTTTCAGATCCGGTACGGAAGACATCTTCAGAATGGCGGAAGC
GATGACCCGTCACACTAGCGAAGTCGACTGCTATCATTTCGGGGAGTATAAAGGCCTCGGTT
CAAGGCACAATGTCCTCCATTGGGGCTGCGGGTGCAAGGAAGCGAGAATTAGCATGGCCGG
CCTTCATAAGCCTTATTACTTTTTGACGGCGGACGAGAGAACGGGAGACGTGTTATCTGAAG
TAAAAGATGCCGATCGAGCGCTTGAGACGCTTGATCCGATGAGAGCTTATTATCCGGAAAA
AGAGTTTCCCGCCCACGCAAGAGTAGGCCCCGACTGGGCGGCGTTCTGTTCCAACTGGCTGA
CGGAATGGGAGCGGACAGGCGATGAAGCGTACCTTGATAAAATCAAACGGGGGATCGGCCG
GCTTAAAGCGCTGCCGTTTCGTCTGTTGTCCGGACCGACTTTCGGATACGATCCTGAAGCTTC
AGATCTGCTTCATATGGGGGACGGGAATCATGGCGGCTATCATATGGTCATCGCCTTCGGTG
CCCCGCAAACATGGCTGGAGCTTGCAGAACTGCTTGAGGATGTGGAATGGAAGGAGATGCT
AGGGGAATTCGGCGAATTTTATTTGCTGACTGATGAAGAAAAGCGCAAAAGGAGCGGCGGG
AAGCTTCACGATGATTTATTTCACTGGCCGATGTTCTCCGCTGCCATGGCCGCTTACGCTGCC
TGGCTCCACCAAGACCGGAAGCTCGCCGAACAAGTGTGGCGCCTGTTGCTCGATGAAAAAC
AAAGCCATACACCGTTGCCGATCACTCCAAAACAAATCCATGCCTGGAAAACGATCACGGA
AATTCCCTGGGTTACAACAAACTGCGTCTCACAATGGTGCCTAAACTTGATAGCCGTGCTCG
AGCTGCTTAAAGATATGCCGCCCGATCTGGACATGCAAGAAAAGATCATCAAGGCAATGAA
AGAACATCCCGCTCGCACA**TGA**

**Protein-19** (WP_020451108.1)

AAAAATCCAACACAAACGCCGCCTGTTCACACGGGCGGCTCTTTTCATGTCCTCAGCCCTTTT
TAGGCCGAAAGAACCGTTATATAAGGTATGTCCGCCCAAAAATCATTAAGACTTTTGATTCA
TTCGTACGATTTCCTTCCGTATCCTTTTCTTTTAACATATTTGTAGTAGATGATGGAAGGGAA
GGAAAATATGTA**GTG**ATTGACGATGGAATAGCGTTAGAACGAAAAATCAAACGAAAAATAT
ATCAGGAAGACATTCACTCTCTTCAGCTATACGTAAAAGAAGTGACTGCCGCCATTGACGAG
CTGAGGCAGGAAAGTTCTTCTATTTTAAAAGCACACCAAATGTATATCAACGGATGGCGCGG
ACAGGCGCGCGAAATGTATGACGCACTTTTGGACGATCTTGACCGGGCGGAATCGCGCGTGT
ATGACAGGCTGAGGGCCATTAAGGAGCAGGCGTCCGAAGAAATTGAGCGGCTTCAGCTGAA
AGCCGAGGAGCTGACA**TGA**

**Protein-20** (WP_020451191.1)

TTCAAGCGGCGCTGAAGAGACAAAAAAATACAAAAGCGAAAATGAACTCTGTTTTATAAAG
ATGAAAACGCGCGATTCCTTAAAAAATCGCGCGTTTTTCTATGAGATATTTGCATTTTCTGT
TCTATTTCGTATAATTTAGACATAAGCCTTGCAACTTTAGGAGGTGATCTAGAAACGAATGG
ATAGGAGAGAAAAA**ATG**GACGACCATAATCATACGAAAGAATTAAAACCGACAGTTGAAA
ACCTTTCAAAAGCGATTTATACAGTGAATCGCCATGCAAAAACCGCGACTAATCCCAAATAC
CTGTATCTTCTGAAAAAGAAAGCTTTGCAAAAGCTGCTTAATGAAGGAAAGGGTAAAAAGG
TAGGCCTTCACTTTTCGAAAAATCCAAGATTCAGCCAGCAGCAGTCAGATGTGCTGATTTCT
CTCGGAGACTACTTTTTTCACATGCCTCCAACAAAAGAAGACTTCGCAAGCCTTCCACACTT
AGGTACATTAAATCATTCGTATCGGAACCCTAAAGCTCATATGTCCTTGAATATAGCGAAAC
AGCTTCTTCAAAACTACACGGGAATTAAGGAAAAACCGCTCGTCACAAACCGGAAGCGCCC
TGCCTCAAAGCCCGTCTTCAAGAAGCTTGGCGAAAGCTATTTT**TAA**

**Protein-21** (WP_026579751.1)

CCGAACCGCTTTTAATATGCCGCCAAACCTTATCAGGCCTGCCCAGGCGTCCTGTCTGAACG
CACATCCTGTGAAAGTCCGGAGAAAGGATGCCATTGCTGACATCGTTTTGTACGTCAGGAAA
AATCTTTTGGGCTTGGATACCGGCTTTGTCGGTAACACTAAGGTCACAAACGATAAGAGCAG
GAGTTGATGTAGGC**GTG**AACGATCAGCTGGCGACAATTCATACCGAACTTCTTCAAATGAA
GGAAGAACTGGAAGCAAGGCTTTTTGAATACTCCTTTTTTCAGCAGGATTCTCAAGGCATCA
ATTCGTATCAGCAGGTAACACTTATGTATCATGTCAAAGAAGAGCTGCAGGACGTGCTTTTG
GCCCTTTCAAAAATCGAAAACGGAACATACGGCATTTGCGAAGAAACGAATCAGGCAATTC
CGCTGGAAAAAATGGCTATTCTGCCGACGGCGCACCGCGAACGATTTTTTATATCATGCC
CAGTTTGAAAAAAAGACGCTTCCTATTTGGGCTGAGACGGATGAGACGTTCAACCGGGCGCT
CTACTCA**TAA**

**Protein-22** (WP_105980957.1)

TCTTCTCAATGAAAGGAAACGGCTACAAAAAAGCAACAGTTGAACCGATAAGAGACGACGA
TTTTTACTTTGACGATAAGTAAGCCGGCCGGGCTAAAAGGGCGCATCCGCGCCCAAGCCCCG
CCTTTCAGTTATAGATACACGGCAAAATGAGAGCAGAACGAATCTGCTCTTTTCACTTTGAG
GAGGAAACGGATACA**ATG**GGAAAAATGGATGAAATCATTCTCGTGGTTCTGCGGGAAAACG
TATTTGCAAATGAAACGCTGACCTTCCAAGGCGTCAACAGCGACGGTGAGAAGGTTGGGAA
GATCATGGCGCATATTGAACAGCACTTCAGTGAAATGAGAAGGGGAGACGCCGAAGAAGAT
CCTGCGTTTAAACAGCCGATCCCTTATGTTGTCATCAAAAGAGAAGACGAAATATTTGTATA
CGAGCGGCTCGAAGGCGGAGGGGAAGCGCGGCTTCACAACAAGCTGTCACTCGGCTTCGGC
GGCCACATGAACTTTGCCGAAGGGCTTGATTTTTCCGGTATTCTGAAGTTAAACACAGACCG
TGAACTTGATGAAGAGCTTCTGATTGAGGAATCAGATAAAGAAAAAATGGTGACGCTCGGA
CTGATTAATGATGATGAAAACAGCGTCGGAAAAGTACATATCGGAATCCTTTCAGCGCTTGA
GTTAAAACCGGGCGCACACGTTGAAGTTAAAGAAAAGGAGCAGATTGCCGGCCGGTGGATG
AAAGCCAGTGATCTGAAAGATAAAAGCGTCTATGACAGACTTGAATCTTGGTCACAGTTTGT
CGTTGATATCTTAGTT**TAA**

**Protein-23** (WP_023857538.1)

CTTCGAATCAAATATTTATGTAAAATCACTCTTTTATTCAAAAACCAAGATCTCTTTCTTGAG
GGGTTTTGCTTTTGAGTGAAATGTAAAAAAGAAGGTATTTCCTCCTATCATGTCGAGAGGAT
TAAATTTTGAAACTTATTCTTTAATTTTCCGTATATCCATATAAAAATGGTATCTAATGCGGG
GAGGAATATTCG**TTG**GCTGAAAAGCAAAAAAGGGTTCCATTTTTTAAAAAATATTGGGTTTC
TCAAATTGGTGTATTTTTATTGTCACAGATAATTTTTATTACATTTGAAACAACAGGATGGAT
ACCTAATTATAGAGAGATTGATGGAACACTATTTGGGAGAATTACGGAATCGTCAATTTTTA
TAGAATGGTTTACTTTTTATGAAACACCACAATTAAATTTACTTACTGTCTTTTTTGGCGTCTT
CTTTCTTGTGCCAGGAATAATAAGTGCATTAAAAAACGTATTTTCGCCTAGACACTATAGTA
ATAGCTCTAAA**TAA**

**Protein-24** (WP_020451915.1)

GACCAATACCGAATAATTTTTTCAGCAATACAGTTGGGCTCCTTTCAAAACGCCTCTTTATGA
TGGCTATTAAAAGAGGAACAGGTTAAATAAATCTTTGGATTCCCCACTATTTAACCACACTT
TCAAATTCCCATCAATGAATAACCATACCAAGAGCCCCTGCATAAGCTGAAAATAAAAAAC
CGGGGTGATGAAGA**ATG**GATTTCAAGGAAAACGACAATCACTCGCTCAGCAAGAAAGACG
AAATTCAAGAAGCGGTTGAACAGTTATTTTACAATTCACCTTTCCACGATATTTTAAATTCAT

TCCAAAAACTGATATCAGACCAGCTTCAGAATTCCACCATTTCCATGAACATCAGAGAGGAG
AATGAGGCGCTTCACGTCGACATTGCCGTTCCTGAATCGTTTCAAAACGGAGATATCATGAT
TGAAGCGAAATCAAGGTATCTTCATGTCGCCTTGAAGGAGAAAAAAGAAAGCGGCGGTTCT
TCATCGTTTACAAGCCTGTCCAGAACCGTTTTGCTCCCATATCCCGTCCGCGAAGAAACAAT
GAAGACGTCGTGGAACCGAAATATCCTCACGGTCTCATTTCCGGAGCAAAAAAAGCATGAA
**TGA**

**Protein-25** (WP_020452052.1)

ACGGGTTTCCTTCCATATCGATCAATTCGACGTCTCCCGTGACCCGGAAACCGCCGTTGTCCA
TCACTTTAATCTGCACTTTTTTGGGCATCATTCCACCTCCGAATCCCTTCATATTTATGCCTGA
CAGTCGGAAACGATGCGCTTTTTCCCGCTCCTGTGGTACAATGTCCGTAAACGAAATAGAGA
AGGATGATATC**ATG**CAGCTTGAAATCACTGCACAGGCAAAACAAGCCATTCAACGGGCAAC
AGAGCCTTATCCTGATAAAAAGATCCAGCTTCGCTATGATTCCGAGGGCTGCGGATGCGCGG
TCAGCGGCGTGCCGGTTCTGTGGCTGACGGACGAATTATCGGGAAGTTGCGAGCCTCTTGAG
ACAAACGGGCTTCCGATTTATATTCAGTCATCGCAAAAAGTCTTTTTTGATGAAGCGATGAC
CATCGACTATTATGAGAAAGCGGGTGCCTTGATGCTGAAAAGTCCGGCACAGGTGCTTTCTC
CCAGAATGAGTATTTTAGTGAAA**TAG**

**Protein-26** (WP_026579290.1)

ATGGCTGAATTGTGCGCTTATGTATCCTTTTTTATGCCGCACTGCTTAAAAAAAGGCTGATAT
CATGTCCATCATTAAATTATAACTGATCCTGCGGAAAAAAACCATCACCCCCGCCCTATTCG
GGCCGGCGGCAAAGGCACTTTGCCACCGTTGGTGCATACCCTATCAAGAGAAGGATTTTGAA
AGGAGGTACAAGT**ATG**TTTCAGCAACAGCCGATGAGAAATCAGCCAGGACCGCAGATGCCG
CAAAGGCCCGGCAGAGCCTTGATGCCGAGACATCCGGGGATGAGAATGCAGCCCCCTCAAG
CGCAGCAGCGCGGCCTGCAGCAAATGTTTCAGCCGATGGGCGCCGCCCAGGAGCAAGCAGG
GGCGCGAGGAGGCGGCGGGATTCAGGGTTTGCTCTCGAAAATCCTTCCTGGATCTGCCGCGT
CCGGGGGAACAGGAGCAGGCCTCGGCGGAACCGGAAGCGGCCTTGGAGGCATTCAGGGTCT
TGCCAATCCGGCTACCATTTCAAACATGCTCGGCAATGTTCAAAAAGCGCTCGGTGTAGCCC
AGCAAGTAACGCCGATGATTCAGCAATACGGACCTCTCGTCCGCAATCTCCCCGGCATGGTG
AAAATGTTCAGACAGCTGAATTCCGATGATACTGAAGCGGAGGACAGCGAACAACAAAAAG
ACGAACCGGACAACAAGACCGAAACGAGCGGCGAAAAGAAAGAAGAAATAAAAGTGTCTT
CCAAAACACCCGAAAAATCCAAAGCCGAAGAAATCAGAAAAAAACGGAGCCGTTCAGCCC
CTCCGTCTGCAAAAAGAAAAACCGGAGGATCAAAACCTAAACTGTATGTA**TAA**

**Protein-27** (WP_020452371.1)

GGGAACCCCGCCTTGCTTTTTGATTAATGTGCTCATTTCATCCGTTTTTCGCGATGCTCCGAT
GGCAATTCTTCTGCCGTTCAATCCTTTTCCCAATCCAATCCCTCTTTTCCGTATCTGTTTCTAT
CAATGTAATGGTTTACTATCATCTTCCAAATGAGATATGATTGAATTATCAAAAAATAACAG
GAGGTTTTTCA**ATG**CATTCTCAATTTGAAACCCGAGCCGAGCATTTGACGAAACATGCCCGT
TTTTTCGAGTATACCACTGCCTCTGACCCGATTGGAAGCGGAATAATCAGTCCGATTCCTGTG
AAAGAGTTCGGACCTGAATTGTATGAAACAGGTGAAACGCGGATCATCCCGCTGGACATCT
CAGAAGAACTGAAGACGGAATATCCCGCTTCAAGCCCTTCCCTTCTCGCCCACTTCATCAAG
ATTCGTTCGGGCGACACGATTCACACCGCCCCCGCGCGACAAGCGAACTATATTACATTGT
CAGCGGCGCAGGGCGTACCGAAGCGGGCGAAGATACGATTCACTGGCAGAAAGGCGATTTT
TTGACTTTGCCTGCCGGTTCAAGCAGCCAGCATACCGCAGCAGAAGATGCAGCCATTTATTA
CATCACCGATTCACCTCTCCTGCATTATTTGGGCGTAAAGCCGTCGGAATCCCGCTTTCAACC

AACCATCTACACCGCTGAACAGGCAAAAGCAAAATTGGCTGAAGCGGCCGCCGCTCCTGAC
GCCCGTTTCCGAAACCGTATTTCCGTTCTTTTAAACAATGATATATTCGATCAAACGCTGACG
ATCACCCATGTATTGTGGGCGATGTTCGGCATCGTGCCTCCCGGCTCCAATCAAGCGCCCCA
CAGCCACAAGTCGGTGGCTCTCGATTTTGTCGCCTATGCCGCACCAGGGACGTACACATTAG
TCGGAAGCAAAATCGATCCGGCCACAAAGGAAATCATCGATCCGAAACGGATTGACTGGGT
AACCGGAAAAGCCTTCATTACACCGCCGGGATTATGGCATTCCCACCATAATGAGTCAGGTG
AAGCGGCCTATATCATCCCCATCCAAGATGCGGGCCTGCAAACATACCTCCGGACACTGGAT
ATTCAATTTCAGCATTATGAAAAA**TGA**

**Protein-28** (WP_234026546.1)

GCCTCCCATTCGCAAATTACAGCTCAAGCACTTGCGGAAAACAGCAGATAATCCGCAGGTA
AAAACGGCCAAGACGTATAGCGTTTTGGCCGTTTTCTATGTTGTTATGATGCTTTTGCCTCAT
GGAAATGCCTTTTTAGGAAAATGTATCGTTTGATCCATGGTTGTACGTTATAATTAATAAAA
CAGGAGGTGATGAG**TTG**AACACTGAATTAAAGGACATGCTTCAATCCGTTCTAAAAGAAGA
ATTGAGTCCGATCAATCAGCGTCTTGATGGAATCGACAAGCGCTTAGACAAAATCGACGCAC
GCTTCGTTGAGGTCGACAAGCGCTTCGATGAAGTGGATGCACGCTTCGCTGAGATCGACAAG
CGCTTCGATGAAGTGGATGCACGCTTCGCTGAGATCGACAAGCGCTTCGATGAAGTGGACGC
ACGCTTCGTTGAGATAGACAAGCGCTTCGATGAAGTGGACGCACGCTTCGTTGGAGTAGATA
AGCGTTTTAACGCAATCGACAAGCGCTTCAAAGAAATAGACGGGCGCTTAAACAAAGTAGA
AAACCGCTTAAACGCAATGGACAAGCGGCTGAATCGGCTTGAAACAGATATAGATGAACTG
AAAAGAGGACAGGAGAGGCTCCCGAAAACGATCATCGAGAACATCGGACAATTTACGGAG
AACATTGTCGAACATGCCGATGATAAGGCGGCTGCCTTGAATGACAGGGTTTTTAGCGTCGA
AACCGCAATACAGAGAATATACAGGTTATCAAGT**TAA**

**Protein-29** (WP_023855527.1)

AAAATTAAAAAATACAACCTCACCTGTCCGAACCAATTCCAAAAAGGCCTTGTTTCAGCCAA
AAACCTCGAAAGCCAGCTCAAATATTGGAGCTGATGCCACTCCTTGCACTTATTCATAAAAA
TAATTGCCAATAACAATTATTTTCTGTAAAATAAAGTTAATAAATGTAATTTTTGTGTTTTTA
AAAAGAAGAAAGG**ATG**AATGAAGAAGTGGAAGCGAAAGCGGCATACGAAAAAGCGGCCG
GGCTCTTGAATCAGTGGTACACCATGATCAAGCGCCATGAAGCGCTCCAGGCTGTATCTTTG
AGATACGAGATCCGAAACCTGCTCTCTAAAATGGAGAAAAACAGAGATTTGGAGCTCTATTT
TCAGCTTTTGGACTACCGCTGCAAATTGATGATGGAGCAGTTTGCAGAATCAGCCGAGCTCT
TCCGGAAGATTAAAAGGCAGAAGGAAACGGTTAAGAACGCGGATGACATCATTCAGTACTA
TTTTTTCTTTTTTTCAGGCATGTATGAGTTTTATGAGAAAAACTATCTTGAGGCGATCAGCTG
TTACAAAAAAGCGGAAATGAAGCTGCATAAACTGACCGATGAAATTGAAAAAGCGGAATTT
TACTATAAGCTCGCGACTGCCTACTACCAAATAGATGATCATTTCAGATCGTTGAACTACTC
CGAAAAAGCGCTCTCACTTTTCAGCAAGCATAAAGAATACATAGACAAAACGATCGGGTGC
GAAATGATTCTCGGGTCGGTTCAGTTTGAACTGTTCCGCATCAAACAAGCCGAGGAACACTA
CGGCCGGGCGCTTGATCAGGCCGTTTCCCTCCAAAACCGGCGAATCATCGGGCTGATCTATC
ATAATATGGGTCTGAACTACGCCAAATGCAGCATGCCGCTTTTGGCGGAGGAGCACTTTAGA
AAAGCTCTTTCAATCGGTGTCCATGAGCAATCGGTCTTTGGCATTAACACCCTTTTTGAGCTG
TCCCACCTCATGTACAAAAACGGTTCTCCCGAAGAAGCGAGACGTCTCTGCAAAGAGGGATT
TACCAGAGCGGCGGAACAAGGGGAGGATGAATACGCGGCGAAATTCAGGCTGATTTTCGCC
CTGTATGATGCGGGCCATCCGCTCGATATTGAACTTTCGCTTGAATACATGAGCGATAAACG
GCTATGGCCTCATGTTGCCGAACTGACAAAAGATATAGCCGATTACTACATGAAGTCCGGCG

ACTATGAAAAAAGCGCGCTTTACCTGGAAAAATCGCAGCATGCCAAGAATCAAATATATAA
AATGAAGGAGGGGCTCATA**TGA**

**Protein-30** (WP_105981186.1)

CCGGGAGCTGTTAAAGGGGGGCCTGTTGTGAATGACTTAATGAAGGCCCTTTATTGTGAAAG
GAAAAAGGACGAGCTTAAATCGCGGCTGCTTAAAATGGGATTTTTTAAAACGCCAGACGGC
CGGCAGCTGTATGAGCTTTCATTAACTGAATTAGACGAAATATTCAAAAAGAAATTGATTGA
AAGGGGAAAATGATC**ATG**GCATTTGTAGGGTTTGAAGAATCAGCAGAGGTGCGGCAGCTGG
CCGAAAGTTTAATCGATGAGCACCACCCGCATTTAAAAGATGCAAAGGAACACGTTGGTTTT
TACATCCGGGAAGGTAACAGCAAATGGGCCGGGAAGGCGAAAAAATGCACGGCCTTTGAGC
GTCATATGACCGATTACATGCTTTTCGTTTTCATCAATAAAGAAGCCTGGAAGACGATGAAC
CAAGAGCAGCGAGCCGCCCTGGTCGATCATGAGCTTTGTCATTTCACCCGTGTGGAATGGGA
GGAACCGGATCCGAATGACTCAAGCAAATGGGTTACTATGTATGGGCCTGGAGAAGATCCT
GACAGTTGGGGGATCCGCGAGCATGATGTTGAAGAGTTTTCGGAAATCATTGAGCGTCACG
GCCTTTGGGAAAAAGGGATTGAATCATTCGCTGCAGCCGTCAGAGAGGCTGACTATCAAAT
GAATATTGATGATGTGCATAGACTTCAAAGGGTGAAA**TGA**

**Protein-31** (WP_105981199.1)

CTTGACATCGTCAAATCGTTCTTCCGCAAACATTTAAAGCCTGGAAAAGCATGAACATTGAA
GGCCGGATATCCGGCCTTTCAGTGTATATTCCTATCTTTTATGAATATTTTCTAAATTTTTTTC
AGTATATCGGTATAAAGTTCACCTTCAGAAAATAAGATGAAAACGCTTACAACAAAACCAT
GAGGTGAACAACG**ATG**AAAAAACTGGTACTGCTCATGCTTGTTTTGCTGGTCGTGTATCCGC
ATGTATCGAAAGCGGGAGGATTTAAAGGAGGAGGAGGCGATCCGGGCCACTGGTTCGCGGG
AGACCCTGTTGAACATCCTGATCCCGCAAAACCGCCGATTGTATTTGTCCATGGATTAAACG
GCTCTTCAAGCGCTTGGTTTGACGAAAATGACATGGCGGAGCAGGCGTGGAAAAACGGATA
TGATGCGGCTTTTATCGATCTCCACCCCGATAAAGACATGCAGGATAACGGAGCGATGCTGG
CCGCGAAACTGCGCGAAATCTACCAGCATTTCGGCCGCAAGGTCATTCTCGTTTCCTACAGC
AAAGGCGGAATTGACAGTCAGTCGGCGCTGATTCATCACAACGCATACCATTATGTCGAGCG
CTTGATTACGCTCGGAACCCCCCATCACGGTTCGCAGCTGGCAGACCTTGCCTACAGCAGCT
GGGCAGGATGGCTCGCCGATATTTTAGGCCAGAAAAATGCCGCCGTTTACTCTCTCCAAACA
GGGTTTATGAAATCGTTCCGCGATCAGACCGACAATCATCCCAATCGATCGAAAACCAAATA
TTTCACCTTGGCAGGCAATAAAATCGGCGGGTTCGGCAGCGTTTTATTTTTTGGAGGCGTCTA
TTTAAACATGTTTGGCGAAAATGACGGTGCCGTCACCGAAAAAAATGCGAGACTCCCTTACG
CCACAAACCTGAATACAGGGAAGTGGATCACTTCTCGATTATTAAAGGAAACCTCACATTT
CCTGTATTTATGCCTTTATTGACGGTTCAGGCATATGCAAATGAAACAGCGGCCATCAAGGA
AAACCTATCGTACCCTTTCATCAGAGGAGGGGAAAATCATGGATTGAGAGAAGAGGAATTC
GCCGTTGAAAAAGGCGTCAAGGAAATAACCGTTCACTGGCTGAGCAATCATTCCTCGGGAA
ATATCAAATTGACAGATCCGCACGGCAAACCGCTCAAAGATTTTTCAAAAGCAAAGACGGC
GGATGTTTTTGAAGGGGGATTCGTCCATTCCGCTGCCATTAAAAATCCGGCAGCAGGTACAT
GGAAGATCGCATCATCTGTCAAACAAAAAGAGGCGTTTTTGTTCATTGTGACGTTCGATTCT
CCTCTGAACCAGCAAATTAAAAACGCAGTCACCAGAGAATCGGCAAATCTTGCGAATGTTA
AGACGTCCGTTCGTTCTATCCGCTATGAAAACGGAAAACAGGCCGAGAAAAAGTCGCTGAA
ACCAGCTTCCATCAATGCGCTGCAAAACAGCCTGTCATTCAAAAAAGCGGGCTTGTATTCGG
TGACGATCGACCTTTCAGGAAAAACGGCAGACAACTCGCCGTTTAACAGGACGATCATCCG
CTCTATTTATGTAAATGACAAAGGGGAGAAGTTTGAAAACAGCCCTTTGTCCGAT**TAA**

**Protein-32** (WP_003185659.1)

ATGCAGTAAAAATGTAACTTTTGCAAGTTGTATAACTAAGGAAAACTTTTTAAAATAGAACC
AAAGCTTTTTCAAGAATTATAATGTGTGCATTTTTTTTGCGGAGAATGTTAGCGCGGTTTGCG
TTCTTCGTCTCTGTTGGTGGAGTCGTCTGACGCTTGCGCTTTTCTTATATATAAATAATAAAT
GGGGGTACACGA**TTG**AAAAGGGCAAGTATTGTGAGAGAGAAAAAATACTATGAATTAGTG
GAGCAGTTAAAAGTTCGATCACAAGACGTTACGTTTTCCGCTACAAAGGCAGTAGGGTTGCT
TATGCTGTTCAGCAGATACCTCGTAAACTACACTTCGGTCGAAAGTGTGGAGGATATCAATG
AGGATTGCGCGGAACTTTATTTCAACTACTTGATGGACAACCACAAGCGGCTCGGCATCAAT
CTGACCGATATCAAGCGGTCAATGCAGCTGATAGGAGATATTCTAGATGTCGAAGTCAATCA
TTACCTGAAAGACTTTTCTTTGTCGAATGTGACGCTTTGGATGAGCCAGGAGAAA**TAA**

**Protein-33** (WP_023857076.1)

TGATGTGTCAATTACATTGCCCGGTAGAAAGAGAAACTGTCTTATCACCAGTACAGAGTTCA
TTTGCATACTCATTCAAAGCATTAGTTCCTGCTAATCAAAATTGAAAAATGAATTGCCGGCTT
TCTTCCAAATCAAAAGGTATATTTTACTGTCGCCCTCATAGATTGGATAGAGAAGTTAAAAA
ACGGGGTGAAAAG**ATG**TTCTACCAATTGAGGTTAGCGGAAATGAAAGACATGAATGCGATC
GAAGCGTTTTTAAAAAAGGCGGGGACAAGCCATAAAGGCATTGAGGAAGCGAAGAGCCAG
TTTATCATGATGGAAGACCCTCCCGATGAAATCGTCGCCTGCCTCGGAATGGAGGAATTTGA
AAACAAAAAAGGGCTGCTGCGCTCGCTTGTCGTGTCTGACAAGCTCAGCCAGGGCCACATC
GTTTCCCTGTTTCAAAGCATGCAGGTATTGTGTGAAAAGCGGGGAATCCAAACCCTGTATTT
AGTTGCCAATAAACGAACATCGATGGATTTCCTTGAAGTGCTCGGTTTTAAACAAGCTGCGG
CAATTCCGGAAGAGCTTGATGGATCAGAGCATGTTTCAGACTCGTTGAGCGTAAACGGAGCC
GTTTTGATGGAGAAAACGTCTAAT**TAG**

**Protein-34** (WP_023856950.1)

TTTGCCGGCAGCCGTTCTGCTGTATTTCCCTTTACATATCATGCATTTTTTAAAAGTTTTTAGA
ATTTTTCTCAAAAAATATTTACTTGAATGGTAAACGGTGATAGATTGATGGAGCAGGAAAAG
GAATATAGTAGATGATGAACTTATTTTTTCCTTTTTCATCTTTTTTAACAACATTCATTAGAA
GGGGGAAGTAC**ATG**TTAAAACGTAAATTTATCGGTAAAATGGGAGTCGGGCTATTAACTTC
AGCAGCATTGTTTTCTTTCATTCTTCCGACGGAGGAAGCGAGCGCCACTTTTTTCAGAAACGC
TCCGACGCTTCATGTCGAAACGGTTGACAGCAACAAAGAGTGGACAACCTCAGACATTGAA
GTCACTTACAAACCGAACTCTTTTGTCGGTGCAAGCTATGTTGAATTTAACTTTCCATACCGC
TTCCATGCGAACACAAGAGATTCGCTGAACGGCAGAACACTGAACTATACACAAATTTTAA
ATGACGGGCAAACCGTCAGAGTGCCGGTTTATGCTTTCAGCTCTTCTGCGTTCAAACTGGTC
ATGGTTCGGAAAACGCTTCCAAACGCAGGAACCCACAGAGTCACTGCGGAGCTGCAAAAGT
TCGGACGAAACTACAACCACGCTGAAGCTACAGTTGATATCCTGCCACGC**TAA**

**Protein-35** (WP_026580354.1)

CGTGGAATAATGCATCATTAAAGGGGCGTTTTTCTTCGGCGAAACATATTTTTTGCTCTCTTC
CGGAATGTCGTATTCTAAGTCGAAGCTAAAGGTATATAAATGTGATTGTAAAAGGTGATGAG
CAATATGTAGGCAATAATTGAGATCCACTTTTCTTTTGATAAAACAGAATTTTTACGATCTGC
GGGTGAAGGACA**ATG**AAGAAAAGATACAGATATTATCGGCTTGATGCTGATACTGTAAATC
AACTGAAGGATATGGACAAGCGCTTGTCAGGACCGCTTCCGGCAGATTTGGATGCGTTTGAA
CAAGACGGGCACGGCTTGTCAGGGGATTTTTACGAGTATTTTGCGCTCGTCGCCGGCAGTTT
AGGTTACGTTTTGGCGGATAAAAAAATGCCTAAGGTGCAACGCCAATCACTAGAAAAAACG

TTTTTTGAAGTTTATCCAAAGAACAAAATCGAAAATTACCCTGAGACTTTTCATGTGGTTGCC
ATGCATGAAAACGTCAGGAGATTGTTGTGTTCGATTGTAAACCGCAGGAAGACT**TGA**

**Protein-36** (WP_023856884.1)

CCTTTGGTATTTTTTTATGAAAATCAATACGAAAATCCTTCTATTGATGGAAAAACATTTTAT
TATTGGACTTACGTCCTCTTTTTGGATAGTCCAAAAAGATCAAAAAAATCTAGCAATCTCCA
TTTTTTCATTTATAATAAGTAAGTCAAAATTATTCTCAAAAAAAACATTCGGGGTGATCTATC
GTGAAAGCACCC**GTG**AGGTATATATGGATTGGTATGATCCTGTGCTTTTTATCGGCCAGTCT
GGCCGTCGGTTGTATCAAAGCTGAAGATCAGTCAGATGATGACAAAAATGATGAAGCGCTG
CAGCCTGCCGAACACTTCGTTTACCATCATTTAATGAGCGATCAGGGACTGATTAAAACCAG
CTTTTCTGACCAGCCTGTTTACTTGTCGGAATCGCTTGGCTTGTGGATGGAATTTTTGATCAG
TAAAAAAGACGGCGAGCATTTTCATGAGCAATATCAGCACCTGACCGAATCCTTCCTGATGA
AAAACAACCTGGTCTCATGGCAAATCCAAAACGGCCAGGCAAGCGGGGTAAACGCCTTGAT
CGATGATCTCAGAATCATGGTGAGCCTCGATCAAGCGGCAGCTCTATGGGGAAACAGCGAG
TATAAACAAACCGCTCTGAACATCGGTTCCGCATTAAAAACGCACAACATGAACAACGGGA
TATTGACGGACTTTTACGACTCCGCCTCTCAATCCGCATCAAAGGATATCACGCTTTCCTATA
TCATGCCAGATGCGCTATCCGTCTTGAAAAAAAACGGAGTGATTGATAAGGATACCGAAAG
GCGGAACGCCAACATTCTTTACCGCGCCCCTTTGAAAAACGGTTTTCTCCCGAAAGTGTACA
GTACAGAAACGAAAGCATACACCTATGACCATGAAGTCAATCTCATTGATCAGCTTTACACA
GCTTGGCATTTACCGCCGAAGGATCAAAAAGCCGCTGTATTAGCGGATTGGCTCAAACAGAC
GTTTCAAACCGGCGGCAAACTGTATGGCCGGTACTCGATCGATACAAAAAAGCCGGCGGTT
CAATACGAATCTCCATCCGTCTACGCTTTGGCGATATTATTTTTCATCAACCAAAACGAAGAT
CAAGACGTCATCAAAGCGCTGTATGATCGAATGAATGATTTTGAAATTCTTGATTCGTCTGA
GACGTATTATGGGGGATATATGAGCGGAAATGATACGCATTCTTTTGATAACCTGCTGCCTC
TATTAGCCGAAAGGAAGCTTTTAAATGAAAATATCATTCAA**TGA**

**Protein-37** (WP_020453535.1)

GAGGCCGGAGGTAAAGCGGGTGTTTCAATTTATTAGAAAAGGCGCGAAAATTCTGCCGTAC
CCGCATTTTTCCGGTTATCCCGGATTTATATTCACAAGCCATCATTCATATGAAGAATGCGCA
GCTTTCTACCGGGAACTGGATGATGAGCTTCATATCATATATCAAAATAATTTGACGGGTAC
AATAGGAGGTTAAG**ATG**ATCTCGACTAAAGTGCAGGAAGAACAAGCCGTGATGATTGTCCA
TACGCTCGGTCCCAAAGGCACGAACTGCGAAAAAGCCGGCCTGCTTTGGCTCAAAAACAAA
GGGGTCCGCGGGGAAGTCAGGCTTTATGAAACGTTGGAAGAAGCGCTCCCTCATGTTAAATC
GACAGAAAATTCAGTTCTTTTAGGCTGTGCCGTTTACCCGTTTTTGCATCAAATTGTATTCGA
GAATCTCGCTGACTTAAAGATCATAGACAGTTTTGTGATGCCGACATACAACATGGTTTTTG
CCGCAAAACAGCACACGATGGCATCTGACATTCAAACCGTCGCTTCACACCCTGCTCCCGCC
CATCTGGCGAAGCTGTTCAGCAGTCAAGTCCAATTTGTCACCAGCAATGCACAAGCGGCCCT
GGCCTGCGCTTCAAACGCGGCTGACGGCTGTATCACGACGATTAAAGCAGCTGATGAACAC
CGTTTGAAAGTCATTCAAGATTTTGGAGAAGTGCCGATGTGTTTTACGATCCACGGGCTTAA
ATCAATCGAAGGAGGAAGCCGA**TGA**
