# Supplementary material for: In silico functional annotation of hypothetical proteins from the Bacillus paralicheniformis strain Bac84 reveals proteins with biotechnological potentials and adaptational functions to extreme environments
Source: PLoS One. 2022 Oct 13;17(10):e0276085. doi: 10.1371/journal.pone.0276085 (PMC9560612; doi:10.1371/journal.pone.0276085)

Figure S3: Ramachandran plots for the 3D models of the 9 proteins by the SWISS-MODEL server

WP_1095290960.1

General

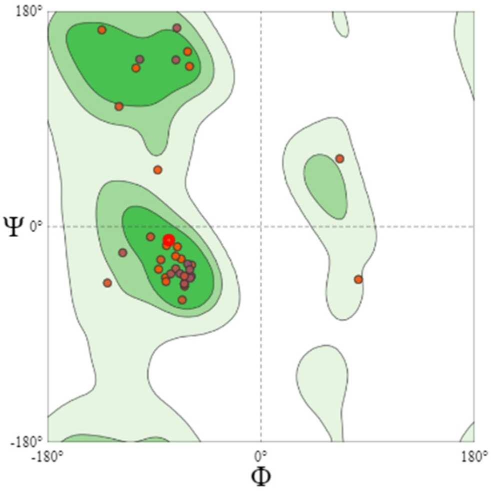

Glycine

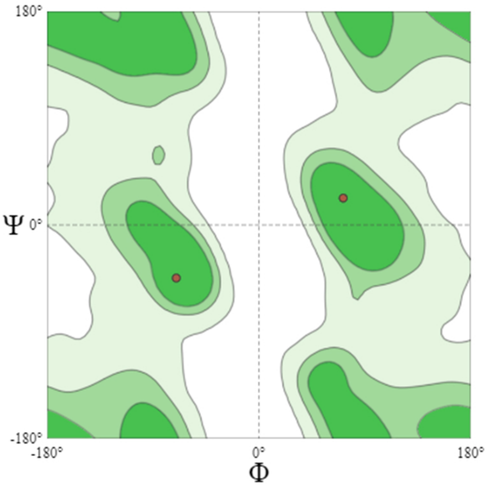

Pre Proline

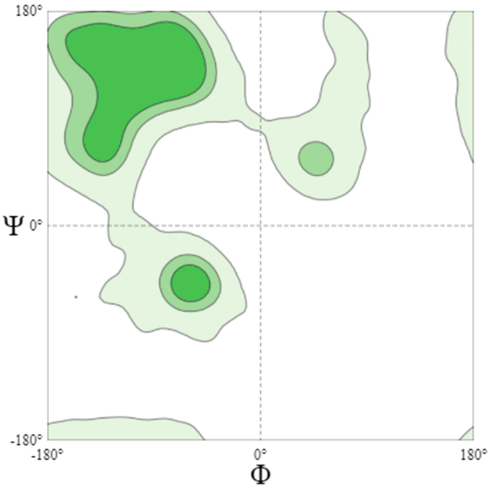

Proline

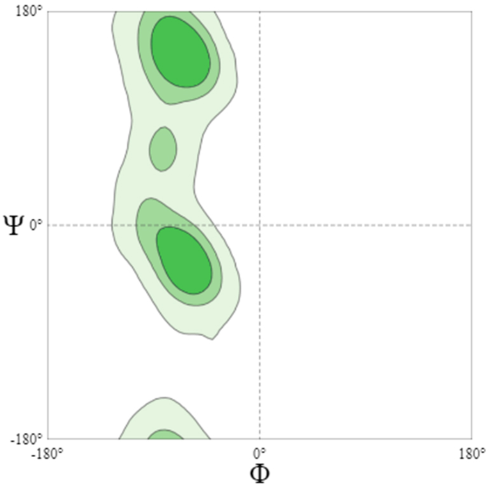

WP_006638778.1

General

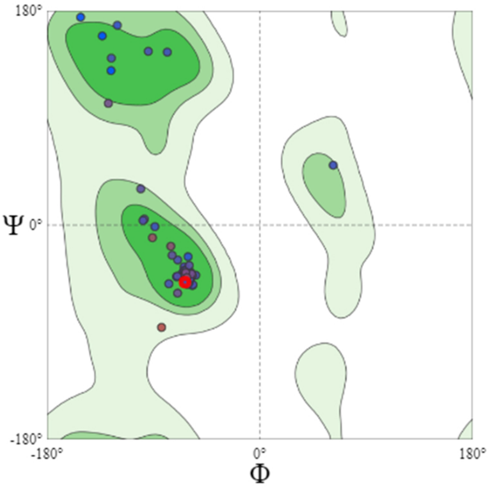

Glycine

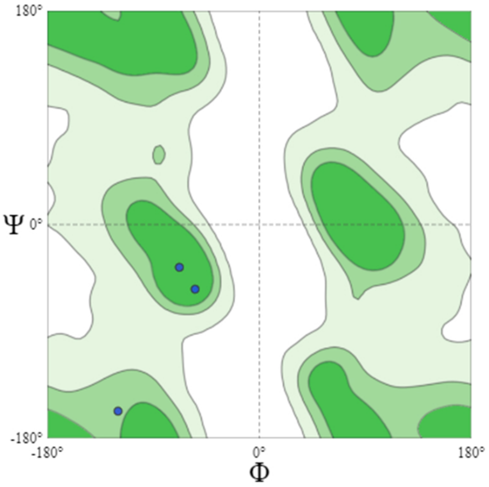

Pre Proline

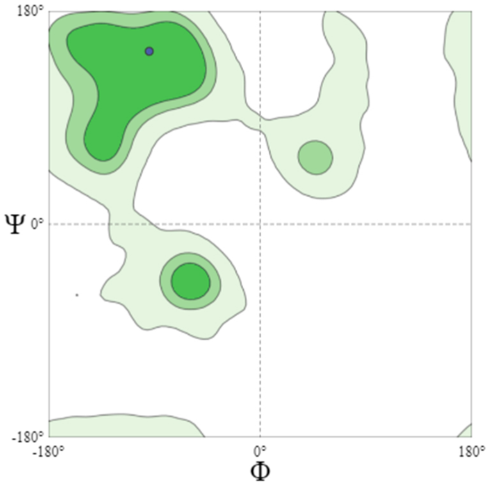

Proline

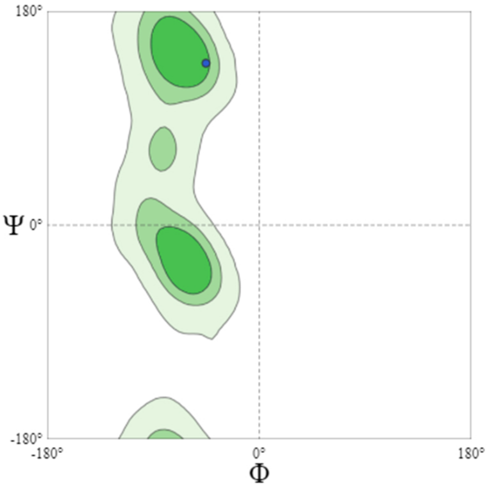

WP_009328837.1

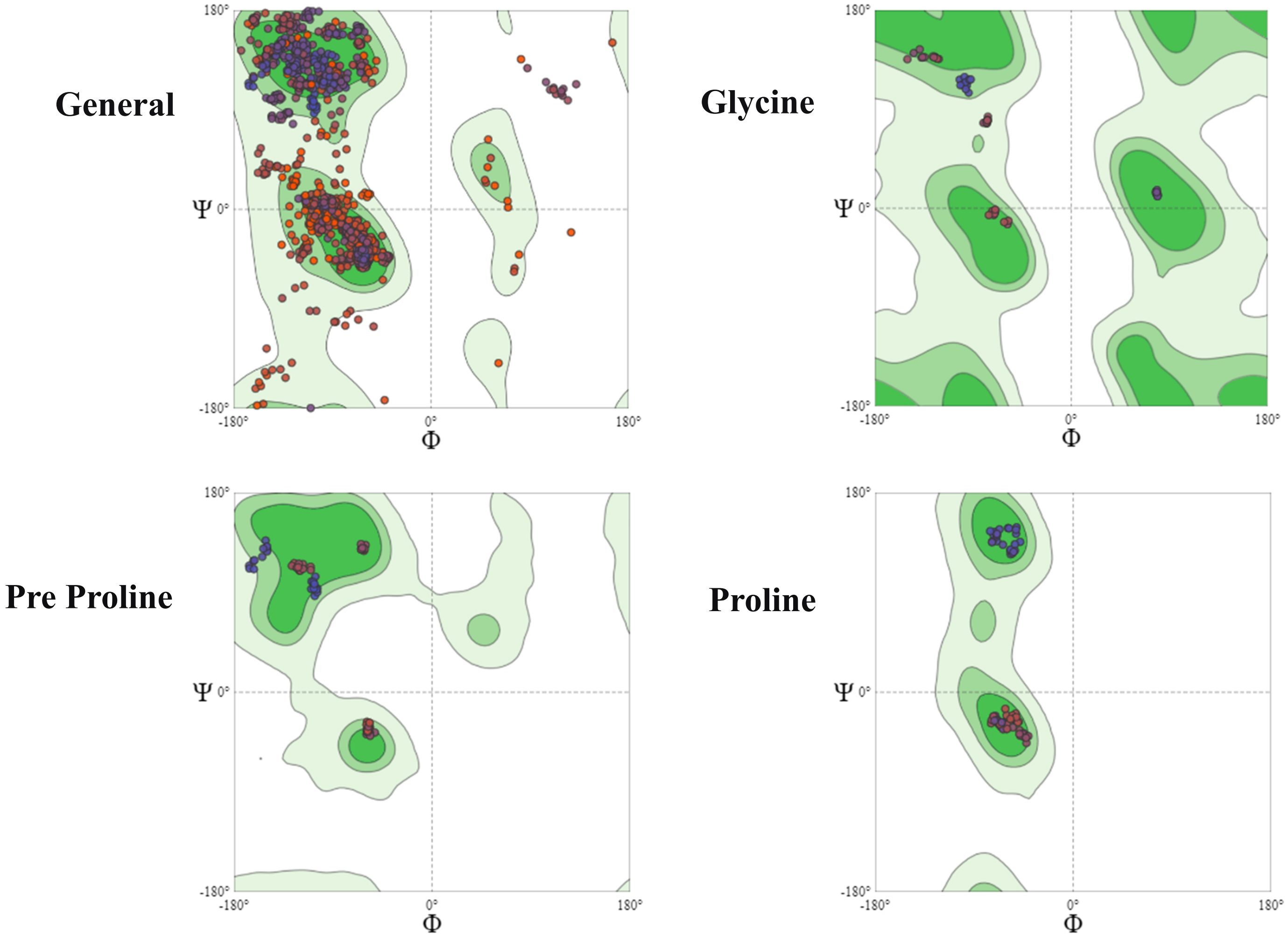

WP_026579751.1

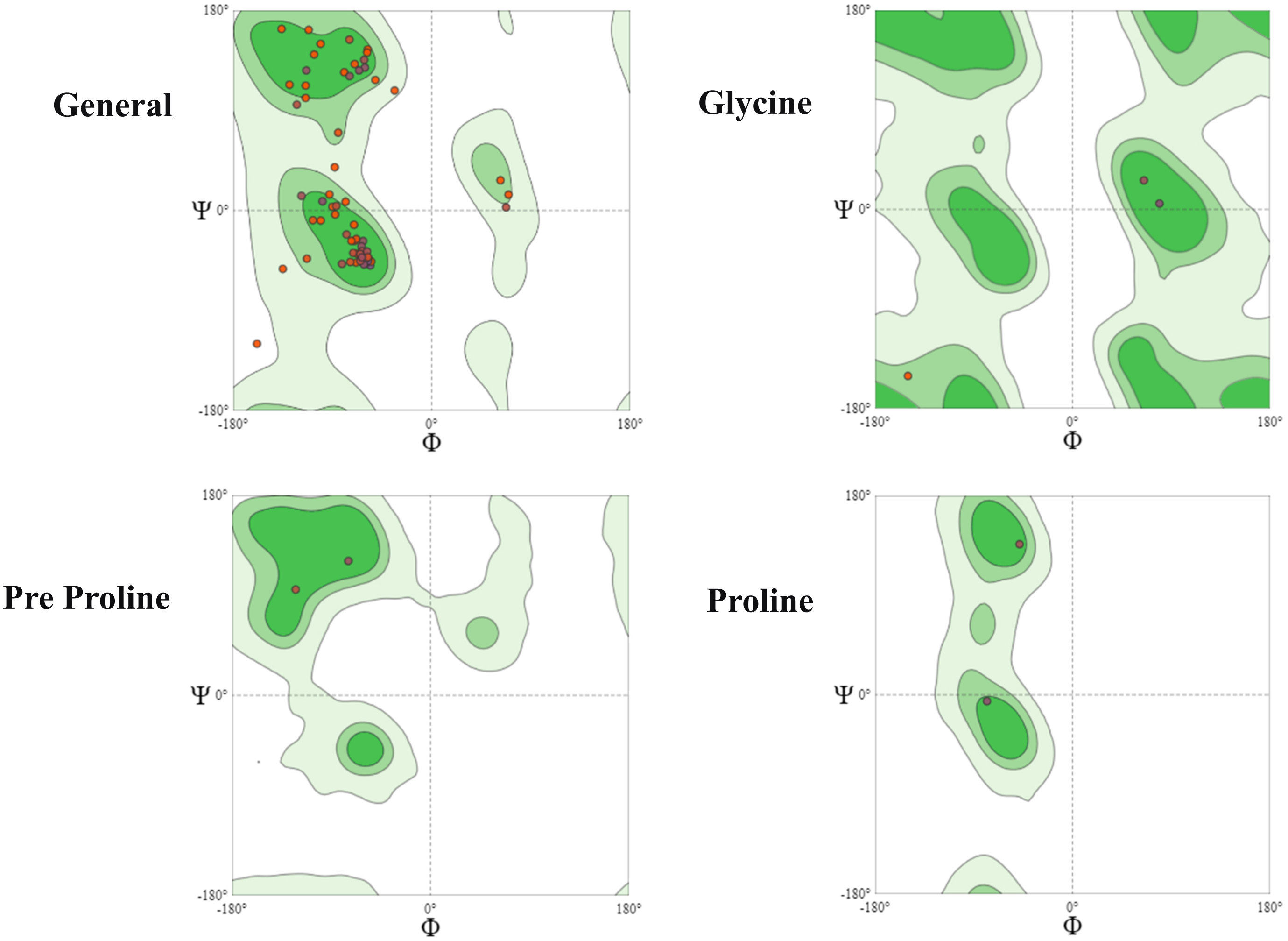

WP_105980957.1

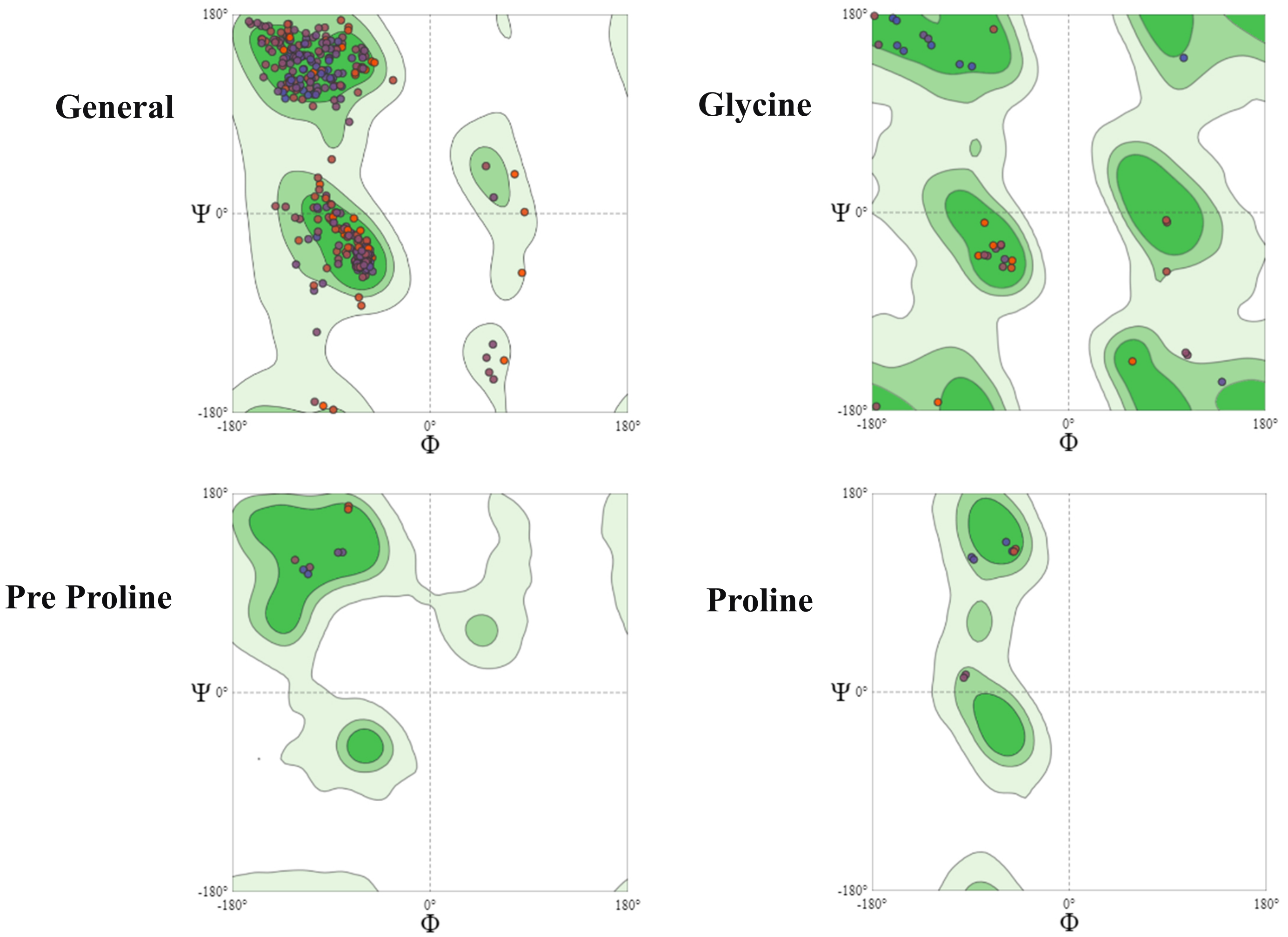

WP_023855527.1

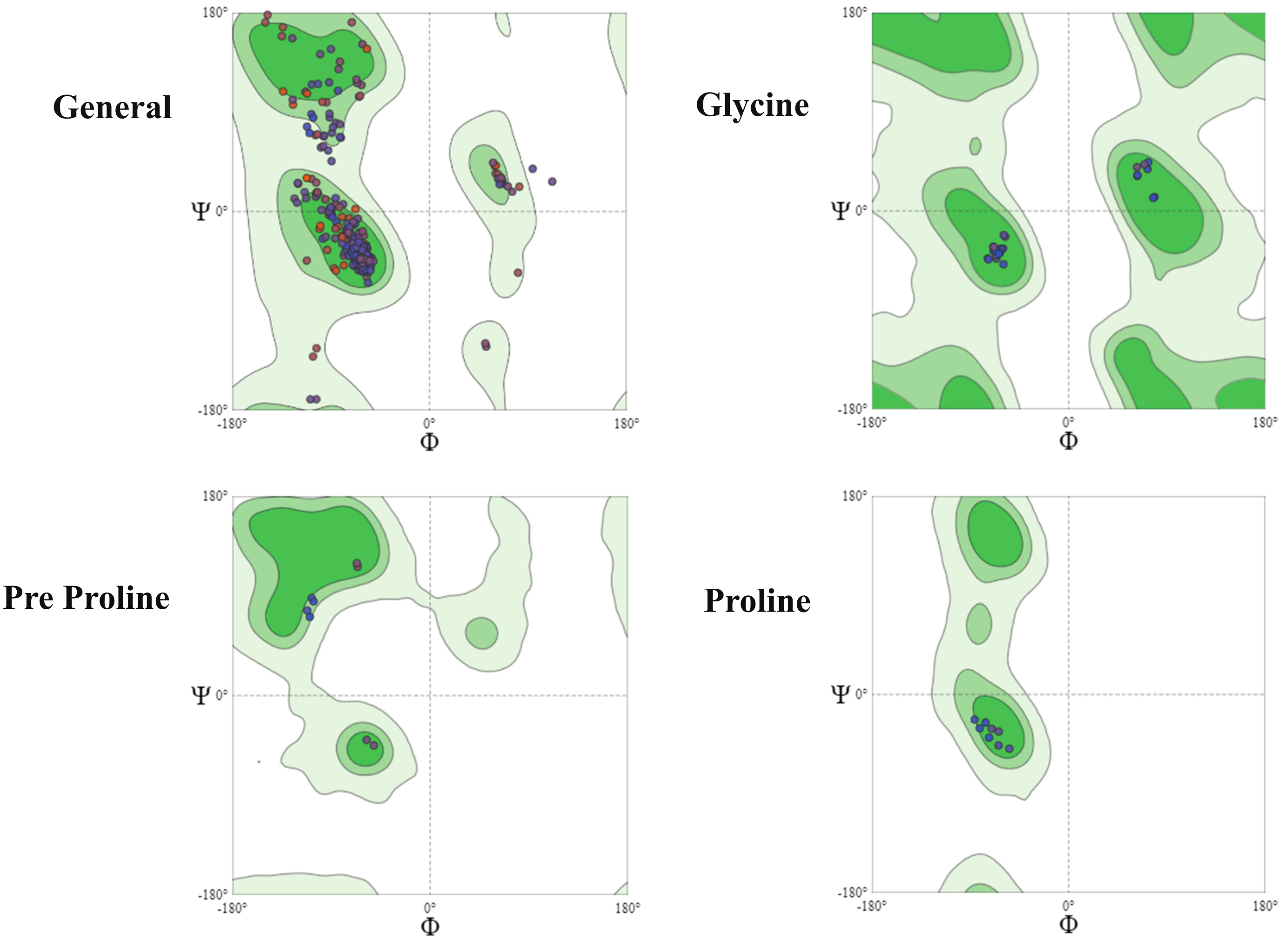

WP_105981199.1

General

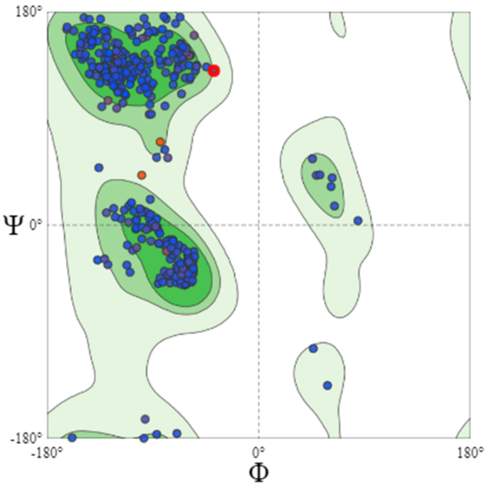

Glycine

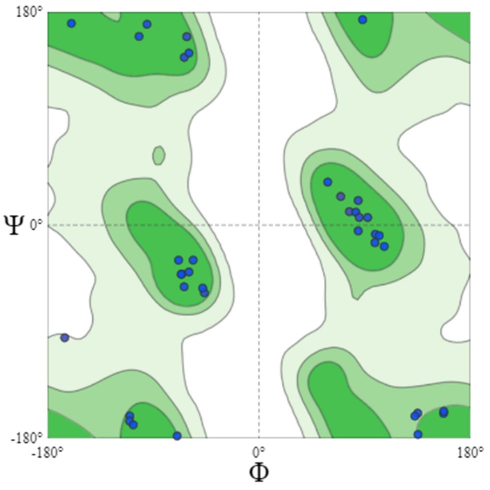

Pre Proline

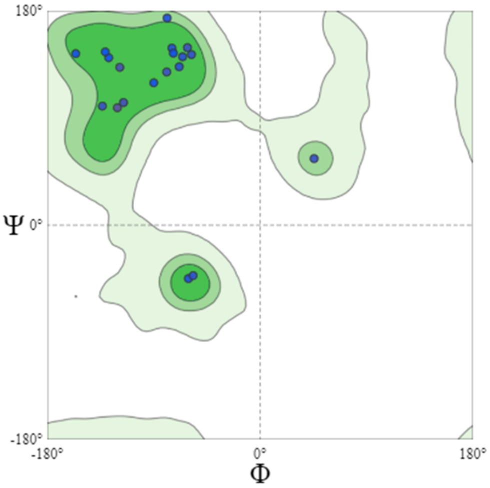

Proline

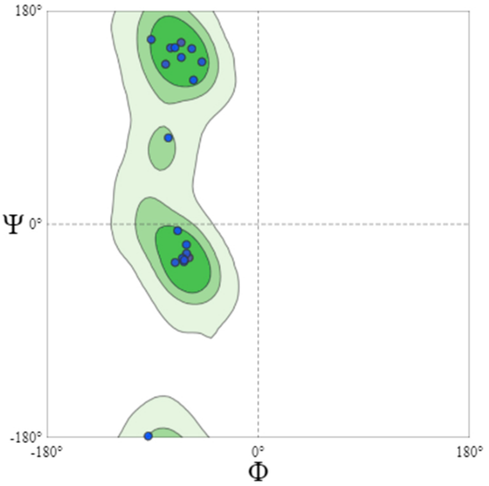

WP_023856950.1

General

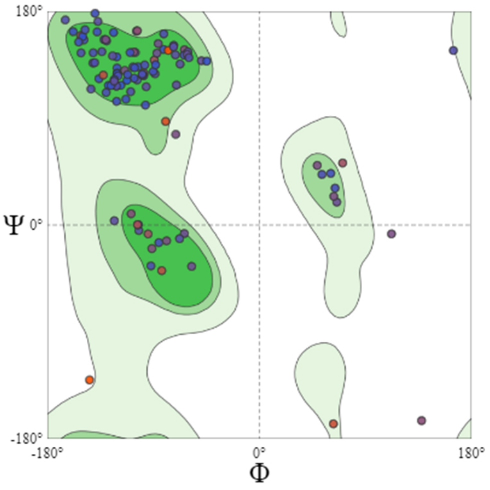

Glycine

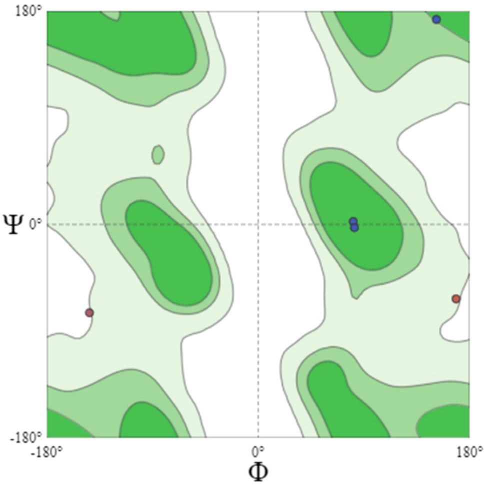

Pre Proline

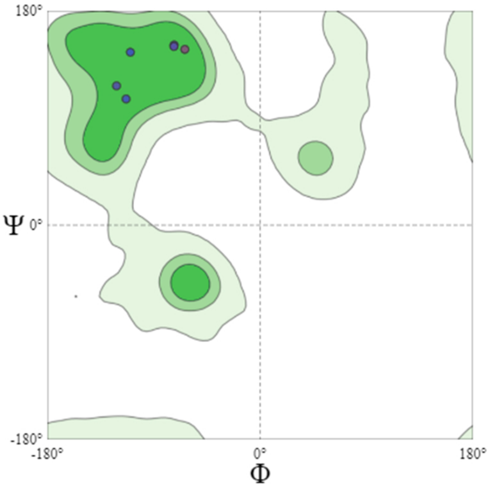

Proline

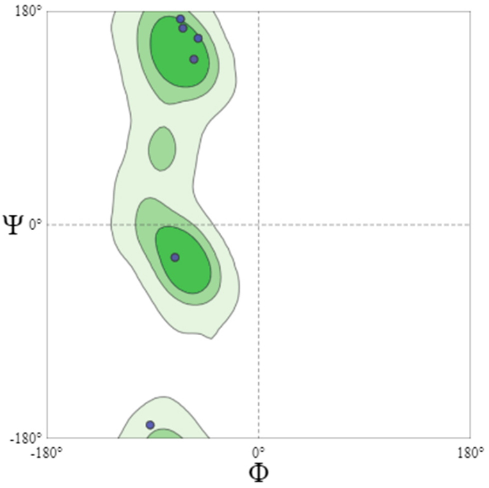

WP_023856884.1

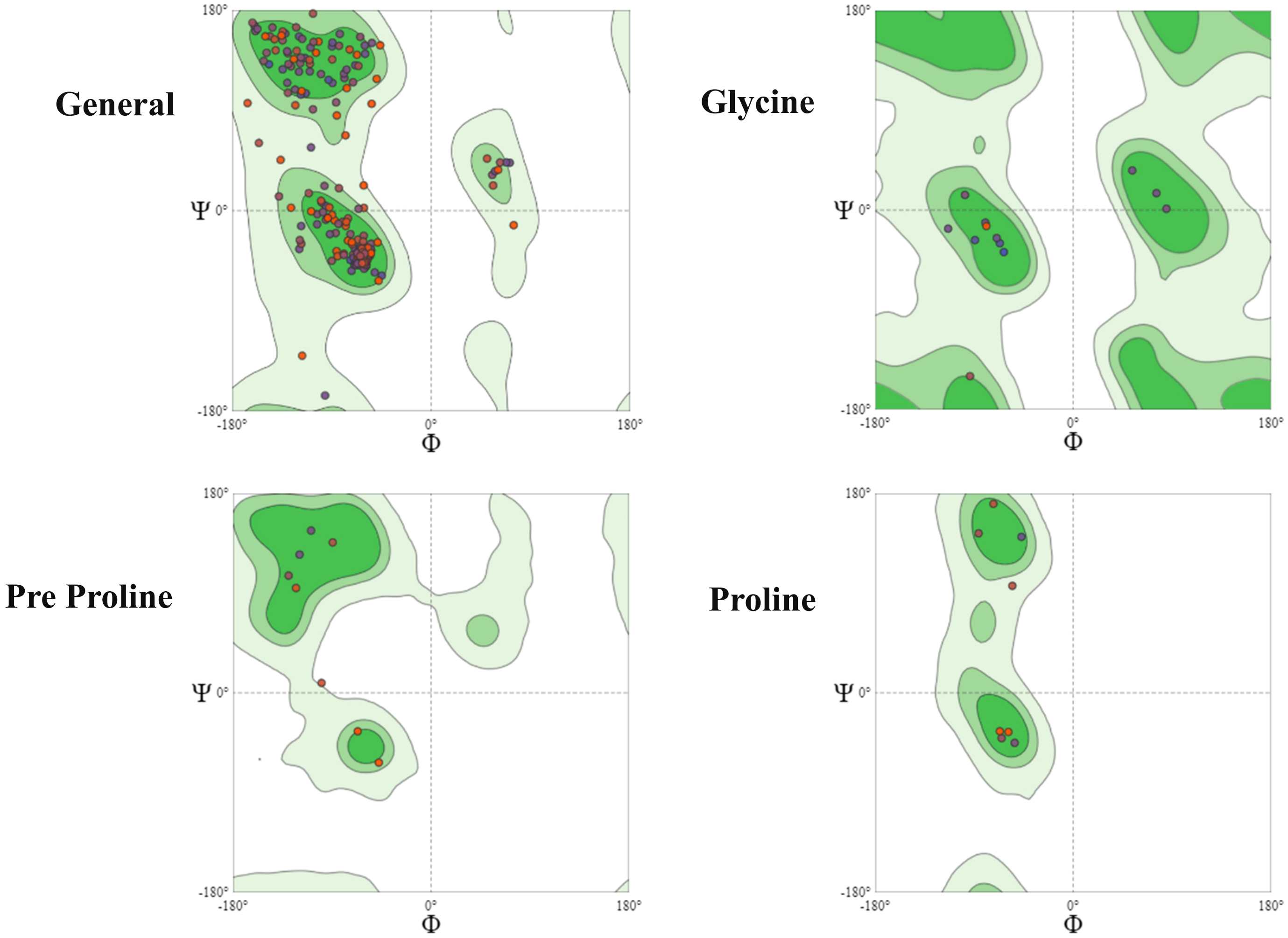

Supplement: S3 Fig — (PDF) [file pone.0276085.s003.pdf]
