# Supplementary material for: In silico functional annotation of hypothetical proteins from the Bacillus paralicheniformis strain Bac84 reveals proteins with biotechnological potentials and adaptational functions to extreme environments
Source: PLoS One. 2022 Oct 13;17(10):e0276085. doi: 10.1371/journal.pone.0276085 (PMC9560612; doi:10.1371/journal.pone.0276085)
Supplement: S4 Fig — (PDF) [file pone.0276085.s004.pdf]

Figure S4: Alignment results from the Superposition analysis.

* Results are obtained from the Chimera MatchMaker.
* The Clustal X color scheme was followed.
* The superposed areas were indicated with red colored boxes.

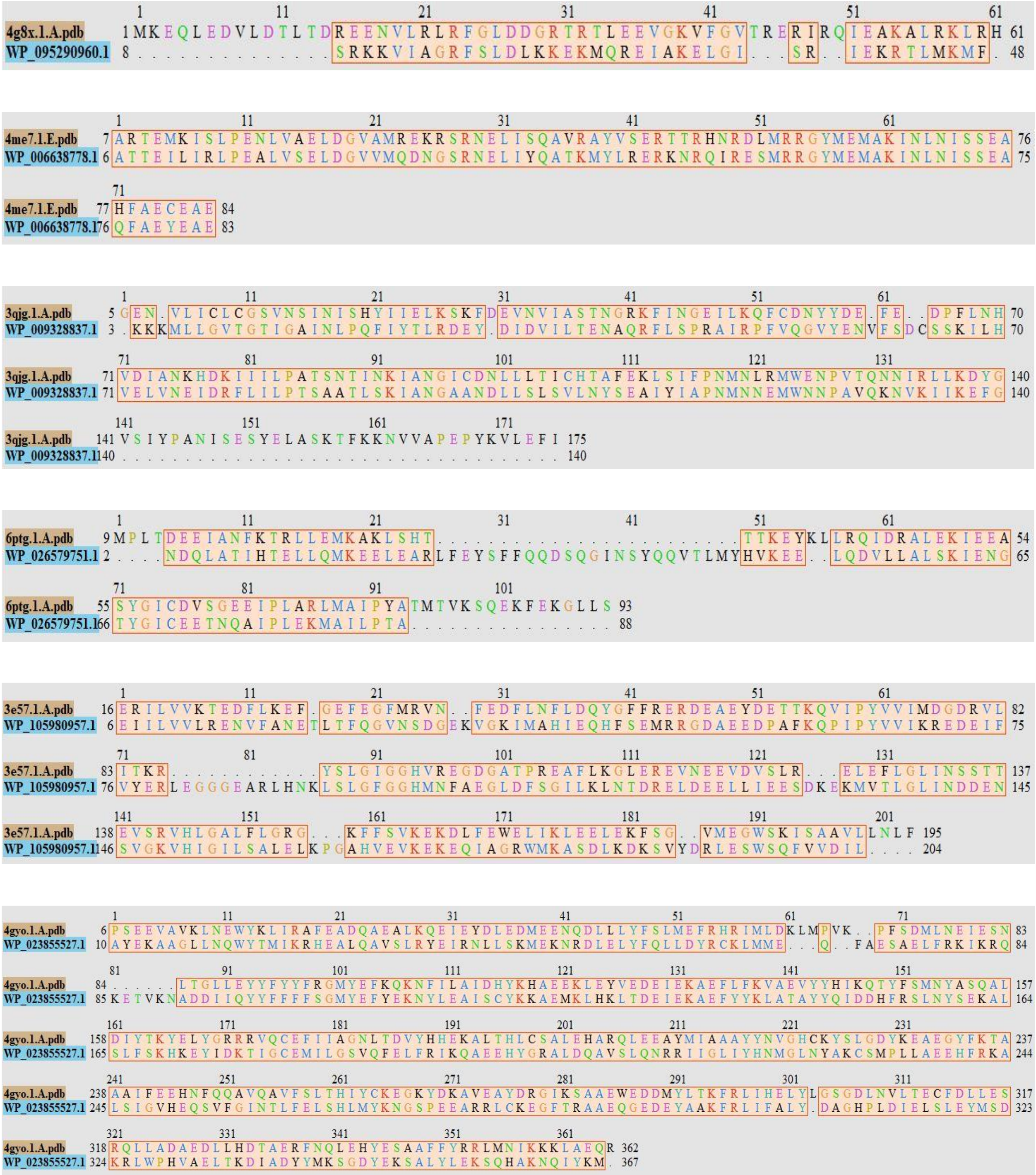

```
                  1         11        21        31        41        51        61        71
6wpx.1.pdb      29 GNPGYWFAGDPVEHPDPAKPPIVFVHGLNGSSSAWFDENDMAEQAWKNGYDAAFIDLHPDKDMQDNGAMLAAKLREIYQY 108
WP_105981199.1  29 GDPGHWFAGDPVEHPDPAKPPIVFVHGLNGSSSAWFDENDMAEQAWKNGYDAAFIDLHPDKDMQDNGAMLAAKLREIYQH 108

                  81        91        101       111       121       131       141       151
6wpx.1.pdb     109 FGRKVILVSYSKGGIDSQSALIHHNAYHYVERVITLGTPHHGSQLADLAYSNWAGWLADILGQKNDAVYSLQTGFMKSFR 188
WP_105981199.1 109 FGRKVILVSYSKGGIDSQSALIHHNAYHYVERLITLGTPHHGSQLADLAYSSWAGWLADILGQKNAAVYSLQTGFMKSFR 188

                  161       171       181       191       201       211       221       231
6wpx.1.pdb     189 DQTDNHPNRLKTKYFTLAGNKIGGFGSALFFGGVYLNMFGENDGAVTEKNARLPYATNLDTGKWDHFSIIKGNLTFPVFM 268
WP_105981199.1 189 DQTDNHPNRSKTKYFTLAGNKIGGFGSVLFFGGVYLNMFGENDGAVTEKNARLPYATNLNTGKWDHFSIIKGNLTFPVFM 268

                  241       251       261       271       281       291       301       311
6wpx.1.pdb     269 PLLTIQANANETAA....LSYPFIRGGENHGLREEEFAVEKGVKEITVHWLSNHSSGNIKLTDPRGKPFKDFSIAKTADV 344
WP_105981199.1 269 PLLTVQAYANETAAIKENLSYPFIRGGENHGLREEEFAVEKGVKEITVHWLSNHSSGNIKLTDPHGKPLKDFSKAKTADV 348

                  321       331       341       351       361       371       381       391
6wpx.1.pdb     345 FEGGFVHSAAIKNPAAGTWKIASSVKQKEAFLFIVTFDSPLNQQIKNAVTRESSNLANVKASVRSIRYENGKQAEKKSLK 424
WP_105981199.1 349 FEGGFVHSAAIKNPAAGTWKIASSVKQKEAFLFIVTFDSPLNQQIKNAVTRESANLANVKTSVRSIRYENGKQAEKKSLK 428

                  401       411       421       431       441       451
6wpx.1.pdb     425 PASINALQNSLSFKKAGMYSVTIDLSGKTADNSPFNRTIIRSIYVNDKGEKFEN 478
WP_105981199.1 429 PASINALQNSLSFKKAGLYSVTIDLSGKTADNSPFNRTIIRSIYVNDKGEKFEN 482
```

```
                   1         11        21        31        41        51        61
5mkd.2.A.pdb      1 QSASIEAKTVNSTKEWTISDIEVTYKPNAVLSLGAVEFQFPDGFHATTRDSVNGRTLKETQILNDGKTVR 70
WP_023856950.1   37 NAPTLHVETVDSNKEWTTSDIEVTYKPNSFVGASYVEFNFPYRFHANTRDSLNGRTLNYTQILNDGQTVR 106

                   71        81        91        101       111       121
5mkd.2.A.pdb     71 LPLTLDLLGASEFDLVMVRKTLPRAGTYTIKGDVVNGLGIGSFYAETQLVIDPR 124
WP_023856950.1  107 VPVY..AFSSSAFKLVMVRKTLPNAGTHRVTAELQKF.GRNYNHAEATVDILPR 157
```

```
                  1         11        21        31        41        51        61        71
6r2m.2.A.pdb    10 SAPKETTPTSTSVQTYVKENYTAKNGLIVDYKNAQEPHYLAESIGLYMEYLVEVNDSKTFQEQVSHLEKNFITEDNFIKW 89
WP_023856884.1  60 .................................SDQPVYLSESLGLWMEFLISKKDGEHFHEQYQHLTESFLMKNNLVSW 106

                  81        91        101       111       121       131       141       151
6r2m.2.A.pdb    90 EATDA..TTTNAIVDDFRITEALYQASEKFSFPSYKKMADKILANTKKYSAEQGVPVDFYDFVHKKKADTLHLSYLNIQA 167
WP_023856884.1 107 QIQNGQASGVNALIDDLRIMVSLDQAAALWGNSEYKQTALNIGSALKTHNMNNGILTDFYDSASQSASKDITLSYIMPDA 186

                  161       171       181       191       201       211       221       231
6r2m.2.A.pdb   168 MQQIN..........YRDKAYLPIQTVNADPFFTEVFQNEQFQYADPSEVNMIDQMLI..AMAYFDENGDVEPNFDNFLQ 235
WP_023856884.1 187 LSVL.KKNGVIDKDTERRNANILYRAPLKNGFLPKVYSTETKAYTYDHEVNLIDQLYTAWHLPPKDQKAAV.L..ADWLK 262

                  241       251       261       271       281       291       301       311
6r2m.2.A.pdb   236 TELASKGKVYARYQRETKKPSSENESTAVYAFLTQYFNKTNQAKNGKITKELLEKMDTSNPETTHFFDYINKEITLKKHH 315
WP_023856884.1 263 QTFQTGGKLYGRYSIDTKKPAVQYESPSVYALAILFF..INQNEDQDVIKALYDRMN....................... 317

                  321
6r2m.2.A.pdb   316 HHHH 319
WP_023856884.1 317 .... 317
```
