# Supplementary material for: In silico functional annotation of hypothetical proteins from the Bacillus paralicheniformis strain Bac84 reveals proteins with biotechnological potentials and adaptational functions to extreme environments
Source: PLoS One. 2022 Oct 13;17(10):e0276085. doi: 10.1371/journal.pone.0276085 (PMC9560612; doi:10.1371/journal.pone.0276085)
Supplement: S5 Fig — (PDF) [file pone.0276085.s005.pdf]

Figure S5: Comparison of the structures predicted by AlphaFold and Swiss-Model.

The final models were visualized using Chimera 1.16 and orientated using the Chimera MatchMaker.

Protein ID	Swiss-Model	AlphaFold Model	Superposed
WP_006638778.1	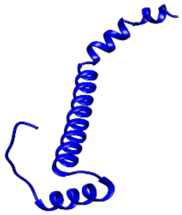	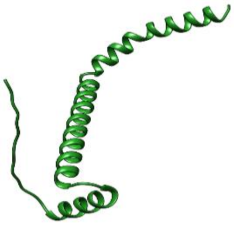	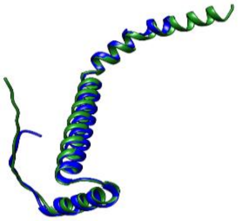
WP_009328837.1	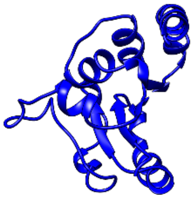	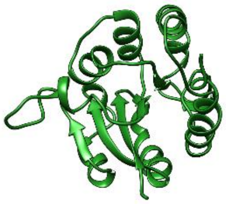	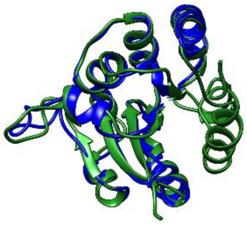
WP_026579751.1	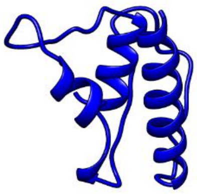	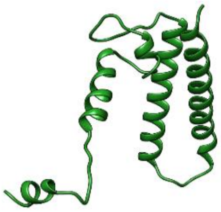	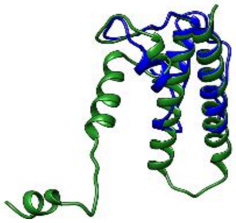
WP_023855527.1	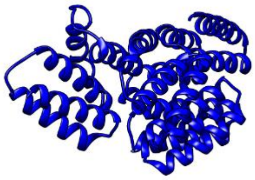	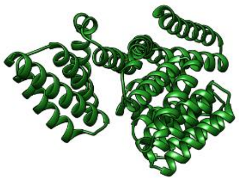	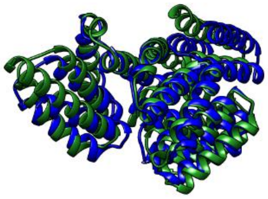
WP_105981199.1	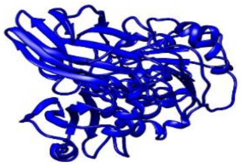	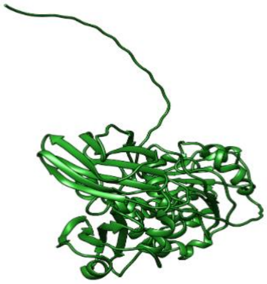	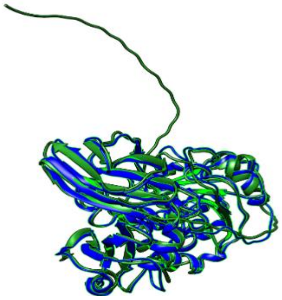
