# Supplementary material for: In silico functional annotation of hypothetical proteins from the Bacillus paralicheniformis strain Bac84 reveals proteins with biotechnological potentials and adaptational functions to extreme environments
Source: PLoS One. 2022 Oct 13;17(10):e0276085. doi: 10.1371/journal.pone.0276085 (PMC9560612; doi:10.1371/journal.pone.0276085)
Supplement: Attachment — Submitted filename: Response to Reviewers.docx [file pone.0276085.s017.docx]

**RESPONSE TO EDITOR AND REVIEWERS**

**Manuscript Number:** PONE-D-22-21684

**Authors:** Md Atikur Rahman^1^, Uzma Habiba Heme^2^, Md. Anowar Khasru Parvez^3*^

^1^ Institute of Microbiology, Friedrich Schiller University Jena, Thuringia, Germany

^2^ Faculty of Biological Sciences, Friedrich Schiller University Jena, Thuringia, Germany

^3^ Department of Microbiology, Jahnagirnagar University, Savar, Dhaka, Bangladesh

* Corresponding author

E-mail: [khasru73@juniv.edu](mailto:khasru73@juniv.edu) (M.A.K.P)

**Paper title:** “In silico functional annotation of hypothetical proteins from the *Bacillus paralicheniformis* strain bac84 reveals proteins with biotechnological potentials and adaptational functions to extreme environments”

**Dear Editor,**

We appreciate you and the reviewers for your valuable time in reviewing our paper and providing constructive and inspiring comments. We strongly believe that the remarks and suggestions have increased the scientific value of the revised manuscript by many folds. We have carefully checked all the comments and tried our best to address every one of them in the revision. We hope the manuscript after careful revisions meet your high standards. The authors welcome further constructive comments if any. We are submitting the corrected manuscript with the suggestion incorporated into the manuscript.

All modifications in the manuscript have been highlighted using the **Track Changes** option. Please find listed below our responses to all comments.

Sincerely,

Md. Anowar Khasru Parvez, PhD

[khasru73@juniv.edu](mailto:khasru73@juniv.edu)

Professor, Department of Microbiology

Jahnagirnagar University, Savar, Dhaka, Bangladesh.

# RESPONSE TO THE EDITOR:

**Editor comment:** After careful consideration, we feel that it has merit but does not fully meet PLOS ONE’s publication criteria as it currently stands. Therefore, we invite you to submit a revised version of the manuscript that addresses the points raised during the review process. While the reviewers were positive about your manuscript, they also raised some concerns and suggestions that can improve its scientific impact.

**Response:** Thank you for coordinating the review process. We have revised the manuscript and addressed all the comments.

**Editor comment:** The quality of all the figures and tables, as well as the figure legends, require to be significantly improved.

**Response:** We revised the figures and tables along with the figure legends according to the suggestions from reviewers and publication criteria.

**Editor comment:** Additional bioinformatic approaches are required to corroborate the structure and predicted function of the modeled proteins from Figure 3.

**Response**: Figure 3 has been replaced by a new figure 3 (A & B) to follow the comments from reviewer 1 and reviewer 3. We have now performed the **structural alignments** between predicted models of several study proteins with the structures of known proteins. Additionally, we have completed a **structural comparison** between several predicted models by Swiss-Model vs AlphaFold to validate the structures and added a new figure S5. We have also presented the protein homology results in Fig S4.

**Editor comment:** Experimental and bioinformatic approaches must be implemented to corroborate if the genes encoding the predicted 37 proteins, a) possess transcriptional in cis-elements and, b) if they are transcribed.

**Response:** Further bioinformatic analyses (promoter and SD sequence analysis) have been included to follow the advice partially and the discussion part has been improved. However, a detailed experimental analysis was omitted, because this would be beyond the scope of this “In silico study”.

**Editor comment:** Several conclusions in the manuscript are not supported by the bioinformatic evidence presented, therefore, these conclusions must be properly adjusted.

**Response:** The points have been properly addressed and revised accordingly.

# RESPONSE TO REVIEWER 1:

**Reviewer #1:**

The work presented by Rahman and colleagues provides an interesting approach for annotating hypothetical proteins in the genome of *Bacillus paralicheniformis* strain bac84. The use of well-established tools is interesting and relevant. The findings, this reviewer agrees that they have both basic biology and biotechnology interests. The manuscript has some issues in the writing that a thorough revision can fix. Overall, this reviewer thinks that the approach is sound and the results interesting. However, in the following lines, I respectfully provide some concerns regarding how the results are shown and described in the manuscript. I hope they are useful for the authors and may help assess important aspects of the study. Overall, the approach reported here is sound, using well-established tools that are valid and relevant for assessing sequence features that may stand out, and thus, assessing the functional role of proteins of unknown function is important.

**Thank you for your time and appreciation. The whole manuscript has been improved, edited, and proofread.**

**Comment 1:** The main concern of this reviewer regarding the manuscript is that the discussion is highly speculative regarding some hypothetical proteins annotated in this study. The discussion can be focused on searching which genes contain a clearer homolog along with good structural comparisons (like structural alignments, authors can either generate the model or use those available in the AlphaFold2 database at EBI) (https://alphafold.ebi.ac.uk/ ) to assess the extent of both homology and relevant residues/domains for each hypothetical protein. This strengthens the workflow used in this manuscript. This reviewer suggests using the metabolic enzymes found, for example, WP_020453535.1, and comparing it with other similar enzymes. Using structural alignments, authors may confirm the fold and critical catalytic residues, thus confirming the success of the analysis.

**Response:** Thank you for pointing this out. We have now performed the structural alignments between predicted models of several study proteins with the structures of known proteins (please see Fig 3A and 3B) and also presented the homology results in Fig S4. These structural comparisons have supported the annotations. For instance, WP_105981199 (annotated as hydrolase) showed high structural similarity with known protein lipase BlEst2. Additionally, we have completed a structural comparison between several predicted models by Swiss-Model vs AlphaFold to validate the structures and added a new figure S5. The results and discussion section along with the methodology have been revised accordingly. Furthermore, the other annotations (WP_025810847.1, WP_020450411.1, WP_020452052.1, WP_023855527.1, WP_003185659.1, WP_023857076.1, WP_003180123.1 etc) were also supported by the BlastP analysis with high identity (≥90%), high query cover scores along with good e-values.

**Comment 2:** Additionally, with my previous comment, I suggest adding in the introduction or in the discussion some features of this particular strain, especially those regarding the known phenotypes it displays, such as biofilm formation, resistance or sensitivity to antibiotics or heavy metals and all that is known about its physiology. Please, check all the available literature supporting the possible role of these hypothetical proteins in the physiology of this strain.

**Response:** We think it is an excellent suggestion. Following the suggestion of the reviewer, we have checked all the available publications and found that this particular strain has potential antibacterial activity against several indicator pathogens. Additionally, a closely related strain has been reported to be involved in the growth promotion of halotolerant plants (auxin production, nitrogen fixation, phosphate mobilization) and another strain can degrade complex polysaccharides such as xylan and lichenan and exhibit activity towards carboxymethylcellulose. We have added these to the revised manuscript with proper references (please see lines 57-63, pages 3-4) in the introduction section. On the other hand, we did not find any possible role of these hypothetical proteins in the physiology of the strain.

**Comment 3:** By checking the hypothetical proteins in Supplementary Table 1, why authors did not discard very short sequences? Can these be annotation artifacts in this and other genomes of close species? Is there any suggestion that these proteins may be real ORFs such as promoter or regulatory sequences? This study will greatly benefit from including the promoter analysis of the 37proteins with a final annotation and support that further experimental validation is worth pursuing. This reviewer also thinks lines 201-202 are unnecessary; why these small proteins should gain focused attention?

**Response:** Thank you for pointing this out. There are many current studies regarding the small proteins encoded from small ORFs (length is less than 50 amino acids). For instance, a hydrophobic peptide called PSM (22 aa) with potential similarities to a-hemolysins has been studied (Queck et al., 2009). Therefore, we did not discard the short sequences. We appreciate the reviewer’s comment on promoter analysis and agree that this is potentially important. We have now performed promoter analysis of the 37 proteins and added the results to the revised manuscript_with Track Changes (please see lines 115-117 and lines 202-210). Proteins with a lower MW (< 10000 Da) need special modifications for analysis in the SDS-PAGE system. Hence, the first few HPs with lower MW require special attention (needs experimental modifications to the standard techniques) to perform further lab experiments. Therefore, we think that the lines are informative.

**Comment 4:** In lines 217-220, the authors make a statement that is hard to follow in the figures presented in this manuscript. I kindly request to provide a figure with the genes involved in response to environmental cues. Also, I recommend toning this statement down since no experimental validation is provided for the hypothetical proteins analyzed here.

**Response:** We think that we failed to explain the point in the original manuscript. We did not give the statement- “The cytoplasmic proteins are in the regulation of several functional processes ....... which may help environmental bacteria to compete with the neighboring organisms in the same ecological niche” **regarding** **our studied proteins**. We tried to show that the cellular localization of proteins is vital for their biological functions in a specific environment and **several cytoplasmic proteins** are in the regulation of several functional processes including biosynthesis, regulatory activities, and transport (Nakashima, H., & Nishikawa, K. (1994)). Therefore, the line has been corrected in the revised version.

**Comment 5:** Several hits (Table S6) seem to be related to phage integrases. Are these proteins in integrons or remanent phages? If they are, this reviewer thinks that additional analysis should be conducted, such as G+C bias with the rest of the genome and codon usage (CAI) analysis to verify if these genes are horizontally acquired and perhaps further analyze them in specific phage databases for enzymatic or structural features more associated with phages.

**Response:** Thank you for this remark. However, we think only one protein seems to be site-specific integrase (WP_230368348.1). The suggested detailed analyses have been omitted because this would be beyond the main objectives of this manuscript.

**Comment 6:** In the conclusion, I also think that the statement that "… this strategy provided us with excellent results…" is too optimistic. Of 414 proteins, 37 are just a fraction of the unknown proteins. I kindly invite the authors to tone down the manuscript.

**Response:** Agreed and changed accordingly.

**Comment 7:** In the tertiary structure prediction section, Swiss-model is a powerful tool, but for some time now, there have been open, collaborative notebooks for using AlphaFold2. In this reviewer's opinion, small proteins should be de novo modelled with AlphaFold2 since recently has been shown to be as effective as NMR structural determinations (Structure. 2022 Jul 7;30(7):925-933.e2. doi: 10.1016/j.str.2022.04.005.). The use of template-based models is very useful for the objective of this manuscript. This recommendation is to validate the structure of at least some of these proteins to prevent bias towards known structures. If the two models are very similar, then confidence in the putative function is stronger. Otherwise, this may result from a forceful prediction of a known template.

**Response:** Thanks for bringing this recent citation to our attention. We have performed a structural comparison between several predicted models by Swiss-Model vs AlphaFold and added a new figure S5 about the findings. The results and discussion section along with the methodology have been revised accordingly.

**Comment 8:** Regarding table 2, the average is not advised here since each tool uses different algorithms to predict and assess conserved domains or features in these proteins. Each tool has its own performance; therefore, the table is informative, but I suggest removing the average, which may be misleading.

**Response:** Thank you and the relevant correction has been performed.

**Comment 9:** The general quality of the main figures is low; they look a bit fuzzy; I recommend using higher resolution images. I think this may have been the result of the generation of the review pdf, but I kindly suggest revising this.

**Response:** We thank the reviewer for the constructive suggestion. The relevant corrections have now been performed according to the publication criteria.

**Comment 10:** Figure 2 legend is poorly described. I recommend indicating the neighborhood of the found proteins and exploiting the data shown here in more detail. Also, Figure 3 provides little information. This reviewer suggests using structural comparisons (alignments) to support their findings further, as mentioned above.

**Response:** We appreciate the reviewer’s comment and agree with it. Following the suggestions of the reviewer, these figures have been improved and Figure 3 (structural alignments) has been added. The improved description for Figure 2 and Figure 3 has been included. please see the relevant amendments on pages 12 and 14 of the revised manuscript_with Track Changes.

## Minor comments:

**Comment 11:** This reviewer suggests modifying the use of "functional annotation" throughout the manuscript. I think the authors should tone down the manuscript since no experimental evidence of these genes is provided. As stated above, I believe this is a good starting point for characterizing these genes. My humble suggestion is to use "predictive annotation."

**Response:** Thanks for the remark. We have done the replacement in several places in the manuscript (mostly in the conclusion). But, we have not changed the title as we consider that the bioinformatic approaches are most commonly used to perform **in-silico** **functional annotation** of hypothetical proteins. Other studies have shown this approach to be correct and used the same terminology [da Costa, Wana Lailan Oliveira, et al.,2018](https://journals.plos.org/plosone/article?id=10.1371/journal.pone.0198965), [Prabhu, D., et al., 2020](https://link.springer.com/article/10.1134/S1062359020300019), [Shahbaaz M, Hassan, Ahmad F (2014).](https://journals.plos.org/plosone/article?id=10.1371/journal.pone.0084263) Therefore, we have preferred to use this terminology. If the editor considers our response incorrect we are of course willing to consider the advice on this matter so the manuscript can be edited where considered necessary in agreement with reviewer 1.

**Comment 12:** Line 2, capitalize *Bacillus*, please.

**Response:** Thank you for your comment. The relevant corrections have been performed. Besides, the entire manuscript has now been scrutinized for solving similar issues.

**Comment 13:** Line 42-43, please italicize B. licheniformis

**Response:** Thank you and the relevant corrections have been performed.

**Comment 14:** Line 44, please change "things" to "products"

**Response:** The line has been corrected in the revised manuscript_with Track Changes.

**Comment 15:** Line 60, please change expressional for expression and function associated data.

**Response:** The line has been corrected in the revised version.

**Comment 16:** In line 61, please change "considerable" with the corresponding percentage of the total coding capacity of this strain.

**Response:** We appreciate the reviewer’s comment. The suggested change has been added to the revised manuscript_with Track Changes (please see line 69, page 4).

**Comment 17:** Line 96, please change to Table S3. Also, please revise the numbering of the Supplementary Tables; S11 is S10.

**Response:** The line has been corrected in the revised version. The numbering of the Supplementary Tables has also been revised.

**Comment 18:** Line 102, please capitalize fasta

**Response:** The line has been corrected in the revised version.

**Comment 19:** Line 114, please correct Line 114, "of a helps to" is not correct.

**Response:** The line has been rewritten to make the point clear.

**Comment 20:** Line 127, please correct to Fig S1. Also, I suggest changing the labels for supplementary Tables throughout the manuscript.

**Response:** Thanks for the suggestion. We have not changed the labels due to following the Submission Guidelines of PLOS ONE for supplementary Tables.

**Comment 21:** Please clarify line 190; RNA interference usually is an experimental approach for eukaryotic organisms.

**Response:** We agreed with the comment and we have rewritten the line to make the point clear.

**Comment 22:** Line 226, I suggest the following modification "…putative RNase (score 0.987) with functional endoribonuclease activity"

**Response:** The line has been rewritten according to the suggestion.

**Comment 23:** Please check gene and protein names to use the correct format; some lack capital letters at the beginning of the name, and genes lack italics.

**Response:** Sorry for the mistakes. Gene and protein names have been checked and corrected where needed in all the text.

**Comment 24:** Line 257, in other places, authors use this statement. I suggest changing to "can be used for biotechnological applications" in the manuscript.

**Response:** The line has been rewritten according to the suggestion.

**Comment 25:** I suggest removing line 280, which is repetitive with this paragraph.

**Response:** The sentence has been removed during the editing of the manuscript and as a result, the sentence is no longer in the revised manuscript_with Track Changes.

**Comment 26:** In line 281, I think "anticipated" is not a correct word here, perhaps predicted.

**Response:** The line has been rewritten according to the suggestion.

**Comment 27:** Please check the grammar in line 328; this line is confusing.

**Response:** The line has been rewritten to make the point clear (Please see lines 372-374, page 19 of the revised manuscript_with Track Changes).

**Comment 28:** Please add italics in line 367

**Response:** Agreed and changed accordingly.

# RESPONSE TO REVIEWER 2:

**Reviewer #2:**

This manuscript employed a structured in-silico approach incorporating numerous bioinformatics tools and databases for functional annotation of hypothetical proteins from *Bacillus paralicheniformis* Bac84 reveals proteins with biotechnological potentials and adaptational functions to extreme environments. The knowledge of these hypothetical proteins' potential functions aids *B. paralicheniformis* Bac84 in effectively creating a new biotechnological target and also facilitates a better understanding of the survival mechanisms in harsh environmental conditions. The findings are meaningful and my comments are as follows:

**Thank you for your time and appreciation.**

**Comment 1:** Functional mining of these hypothetical proteins is interesting in *B. paralicheniformis* Bac84. Are all these 37 hypothetical proteins existing in the species B. paralicheniformis? Which of these hypothetical proteins are specific in strain Bac84? Are these 37 hypothetical proteins can be used as indicators for the classification and identification of the two species *B. paralicheniformis* and B. licheniformis? I think the authors could add these contents into the Results or Discussion parts of this manuscript if possible.

**Response:** Thank you for pointing this out. We mentioned that all the studied proteins are found in the *B. paralicheniformis* strain Bac84 genome and many are found in multiple species in the *Bacillus* genus including *B. licheniformis* (please see Table S1 and S6). We have also found that only protein WP_095290960.1 is highly specific to *B. paralicheniformis* strain Bac84 (Table S6). However, there is no experimental evidence for the expression of these proteins yet. Therefore, we think that these 37 hypothetical proteins can not be used as indicators for the classification and identification of the two species *B. paralicheniformis* and *B. licheniformis*.

**Comment 2:** I wonder if the authors considered whether these hypothetical proteins can actually be expressed. RNA-seq or RT-PCR technologies could be applied to further understand the expression patterns of these hypothetical proteins.

**Response:** We thank the reviewer for this careful thought and constructive suggestion to improve our paper. **Further bioinformatic analyses** (promoter and SD sequence analysis) have been included to follow the advice partially and the **discussion** part has been improved. However, a detailed experimental analysis was omitted, because this would be beyond the scope of this in silico study.

**Comment 3:** The English writing of this manuscript could be further improved. Some small errors should be changed, for example, in line 2 and 6, “bacillus” should be changed to “Bacillus”; in line 37, the word “strain” should be standardized; in line 42 to line 43, “B. licheniformis” should be changed to “B. licheniformis”.

**Response:** We thank the reviewer for these constructive suggestions. The relevant corrections have now been performed. Besides, the entire manuscript has been improved for solving similar issues.

# RESPONSE TO REVIEWER 3:

**Reviewer #3:**

Atikur Rahman et al., analyzed in silico 414 sequences of hypothetical proteins from *Bacillus paralicheniformis* strain bac84 with the aim of attributing, at least in a predictive form, its functional role based on sequence and structure homology with well-known proteins. 37 hypothetical proteins resulted with an associated function before the bioinformatic analyses using several online tools. The authors also mentioned in this manuscript the potential of their findings to possibly use some of these proteins in biotechnological applications. This draft could be recommended for publication in PLoS ONE not without first addressing several points of concern detected.

**Thank you for your remarks. The whole manuscript has been improved, edited and proofread.**

**Comment A)** In figure 1, outlined green boxes are depicted to indicate several steps performed as part of the general protocol applied to all sequences, however, it is not clear why some boxes were green outlined and not others. I assume that tasks described in the green outlined boxes were only applied if the HPs analyzed fulfilled the same predicted function in at least three bioinformatics tools. This is an elementary observation but it could be confusing when interpreting the figure. I suggest that a figure caption with information that allows understanding of the content of the figure be added since the one described in line 99 is only limited to giving the figure a name but does not explain its graphic content.

**Response:** Thank you so much. We have added detailed information in the caption to make it easier to understand figure 1.

**Comment B)** Results of gene ontology shown in figure 2 are confusing to my eyes. What is the rationale to use the “bubbles” representation? What X axis indicated? If the distances between the bubbles do not indicate something significant and it is only to increase the area of the bubble based on the number of proteins that have a certain predicted function, I suggest that the data be represented in another way, for example, in a bar graph (as in section A), a pie chart or even in tables. This applies to items B and C of this figure.

**Response:** Apologize and we have rewritten the figure description to make it easy to understand as reviewer-1 also suggested it. But, we preferred the bubbles representation since it has been used in several previous studies to describe the GO terms distributions ([da Costa, Wana Lailan Oliveira, et al., 2018](https://journals.plos.org/plosone/article?id=10.1371/journal.pone.0198965)) and ([Imam, Nikhat, et al., 2019](https://www.sciencedirect.com/science/article/pii/S2405844019363947)).

**Comment C)** Figure 3 is pretty poor. It is not very informative as it is only limited to showing the structures of the eleven modeled proteins and does not provide relevant information about the findings. If the reader wishes to know what theoretical function each protein has, and whose predicted structure is shown, it is necessary to check the supplementary table S10. The latter is not at all practical when reading the manuscript. As in the other captions of figures 1 and 2, the caption of figure 3 is very insignificant because it does not provide information on the structures, how they were modeled, the selection of colors, and the reliability of the models presented, among others. Please consider these aspects to improve this figure.

**Response:** Thank you for pointing this out. We have now performed the structural alignments and changed the caption of the figure. Besides, the new Supplementary figure S2 (the Ramachandran plots) has now been added.

**Comment D)** Lines 180-181: Please name at least three types of DNA-Protein interactions in the biological context.

**Response:** Lines have been rewritten in the revised manuscript_with Track Changes (please see lines 195-197).

**Comment E)** Line 230: S2 Fig must be S1.

**Response:** The line is corrected in the revised version.

**Comment F)** In lines 244-245 the authors claim: “…proteins with similar sequences usually exhibit similar functions. Proteins dissimilar to current PDB entries may correspond to novel functions.” Can the authors do a more detailed analysis by comparing the structures of known (crystallized) proteins with the structures they modeled? Thus, it would be possible to have a clearer idea of how similar the three-dimensional structures are, as it is mentioned that a part of the modeled protein may resemble the already known one, but another part may not.

**Response:** We think it is an excellent suggestion. Following the suggestion, the structural alignments presented in Figures 3A & 3B have now been included (please see the figures).

**Comment G)** Lines 246-247: Can the authors be more specific about what they consider an "excellent degree of reliability? Table S10 shows the percentage of a favored Ramachandran structure, however, it gives little information on specific Ramachandran conformations for helices and B-strands. A supplemental figure showing each Ramachandran plot for at least the 11 modeled protein structures of Figure 3 might be beneficial.

**Response:** Thank you for pointing this out. We have added a new supplemental figure (Fig S2) showing the Ramachandran plots for the 11 modeled protein structures. We considered the models were highly reliable due to the good GMQE scores along with high coverage, % of identity and Ramachandran Favored percentages of more than 90%. But, we have now toned down this line in the revised manuscript_with Track Changes.

**Comment H)** Lines 248-254: “ROC” is not defined in the manuscript. Please define it. PLoS One is not a bioinformatic specialized journal, therefore, some terms may be unusual for different readers.

**Response:** We have not altered the manuscript since we have already defined the “ROC” in the paragraph “Performance Assessment” (see lines 149-157 in the revised manuscript_with Track Changes). If the editor considers this to be an insufficient response we are of course willing to edit the manuscript so that the description of the ROC analysis is clearer."

**Comment I)** Line 263: a connector is necessary at the end of the line.

**Response:** Line 263 has been corrected in the revised manuscript_with Track Changes.

**Comment J)** Do the authors consider it really relevant for the study to present the information in Table 2? Bioinformatic approaches used are all previously verified and properly referenced here. I suggest eliminating or including it as supplementary material.

**Response:** Yes, we consider that Table 2 is informative. It has been changed in agreement with **comment 8** from **reviewer 1.** We have therefore not deleted or moved it to the supplement section. We consider our approach to these conflicting requests to be the most suitable. If the editor considers this the incorrect approach we are of course willing to follow reviewer 3.

**Comment K)** Figure S1 shows the protein-protein interactions that resulted in the analysis performed on STRING, however, it is not specified what type of hypothetical or experimentally verified interaction was found. STRING provides information in a color code depending on the type of interaction (hypothetical, known, direct interactions, expression, operon array, among others), however, this information was omitted for the results shown using Cytoscape. I encourage authors to consider mentioning this information.

**Response:** We thank the reviewer for this careful thought and constructive suggestion to improve our paper. We have now revised Figure S1. The types of interactions have been indicated with the defined colored edges (STRING analysis).
